# Effects of Combined Basal and Bud-Stage Foliar KH_2_PO_4_ Application on Vegetative Growth, Floral Performance and Nutrient Partitioning in Potted *Hydrangea paniculata ‘Limelight’*

**DOI:** 10.3390/plants15213306

**Published:** 2026-10-26

**Authors:** Shuju Huang, Shizhen Cai, Qianhe Jing, Gaoshuai Hu

**Affiliations:** College of Landscape Architecture, Sichuan Agricultural University, Chengdu 611130, China

**Keywords:** monopotassium phosphate, mineral nutrient partitioning, photosynthetic physiology, flowering trait, root plasticity, transcriptomics

## Abstract

Phosphorus (P) and potassium (K) are essential mineral nutrients for plant growth, development, and reproduction, and their supply levels and application methods may affect the growth, physiological performance, and flowering quality of ornamental plants. In this study, one-year-old potted cuttings of *Hydrangea paniculata ‘Limelight’* were used in a two-factor completely randomized design. Four basal application rates of KH_2_PO_4_ (50, 100, 150, and 200 mg P_2_O_5_ kg^−1^ substrate) were combined with foliar application of 0.1% KH_2_PO_4_ at the bud stage. Root morphology, biomass allocation, photosynthetic characteristics, antioxidant physiology, flowering quality, and the accumulation and allocation of N, P, and K among plant organs were evaluated. Transcriptome sequencing and quantitative reverse transcription PCR (qRT-PCR) validation were also performed for representative treatments. The results showed that treatment 2KF, which combined basal application of 100 mg P_2_O_5_ kg^−1^ substrate with foliar application at the bud stage, exhibited a favorable balance between physiological performance and flowering traits. The maximum quantum efficiency of PSII (Fv/Fm) in leaves was 0.76, significantly higher than that in treatments 1KF and 4KF. The leaf soluble sugar content reached 13.12 mg g^−1^ fresh weight (FW), whereas the malondialdehyde (MDA) content remained relatively low. The root-to-shoot ratio was 1.56 ± 0.48, 38.1% higher than that of the control (CK) and higher than those of the other treatments. Transcriptome analysis identified 8142 differentially expressed genes (DEGs) between 2KF and the nitrogen-only control (CN), including 3386 upregulated and 4756 downregulated genes. These DEGs were primarily associated with nutrient transport, photosynthesis, carbon metabolism, antioxidant responses, and stress responses. In treatment 2KF, the expression of several genes associated with P and K transport was upregulated, whereas the expression of genes related to the phosphate starvation response was suppressed. The expression patterns of 12 candidate genes examined by qRT-PCR were generally consistent with the RNA-seq results. In conclusion, under the conditions of this experiment, basal KH_2_PO_4_ application combined with foliar application at the bud stage, particularly treatment 2KF, produced favorable overall responses in *Hydrangea paniculata ‘Limelight’*. These findings provide a reference for optimizing fertilization management in this ornamental cultivar.

## 1. Introduction

*Hydrangea paniculata ‘Limelight’* is an important summer-flowering woody ornamental shrub. It develops flower buds and flowers on current-year shoots, and its large inflorescences and extended flowering period contribute to its high ornamental value. Consequently, its use in commercial potted plant production and landscape planting has continued to increase [1,2]. ornamental plants becoming increasingly standardized and precision-oriented, optimizing nutrient supply strategies, improving fertilizer use efficiency, and maintaining a balance between vegetative growth and flowering performance have become important considerations in the cultivation and management of *Hydrangea paniculata ‘Limelight’*.

Phosphorus (P) and potassium (K) are essential macronutrients for plant growth and development [3,4]. Their supply levels and application strategies may influence plant growth, photosynthetic physiology, nutrient accumulation, and floral traits, making them particularly important for flowering ornamental plants. However, plant responses to P and K supply are influenced by substrate properties, fertilization rates, growing conditions, and developmental stages. Therefore, fertilization effects observed in other plant species cannot be directly extrapolated to *Hydrangea paniculata ‘Limelight’* [5]. To date, further research is needed to compare combined KH_2_PO_4_ supply at different application rates and through different application methods in the potted production of this cultivar. P is involved in the synthesis of nucleic acids, ATP, and photosynthetic membranes and provides an essential biochemical basis for flower bud differentiation. P deficiency can inhibit root elongation and reduce carbon assimilation efficiency, whereas excessive P supply may induce ionic antagonism with micronutrients, thereby impairing nutrient uptake [6,7] K activates numerous key metabolic enzymes, regulates stomatal movement, and facilitates the translocation of photosynthates from source leaves to sink organs, including inflorescences. K deficiency can impair assimilate transport, restrict floret development and substantially shorten the flowering period, thereby reducing the ornamental value of flowering plants [8] Previous studies have shown that changes in P and K supply can affect root architecture, photosynthetic performance, and plant growth and development across different species, although the responses are dependent on plant species and growing conditions. P and K perform distinct yet interrelated functions in plant physiological processes; therefore, jointly modulating their supply levels may influence vegetative growth and reproductive development. However, whether combined P and K supply can improve growth, photosynthetic performance, and floral traits in *Hydrangea paniculata ‘Limelight’* remains unclear. In addition, changes in nutrient uptake and allocation among plant organs under different P and K supply levels require further evaluation through targeted experiments.

*Hydrangea paniculata ‘Limelight’* flowers on newly developed shoots, making nutrient availability during shoot emergence and subsequent reproductive development potentially important for coordinating vegetative growth with inflorescence formation [9]. During the production cycle examined in this study, plants resumed vigorous growth after winter dormancy, initiated shoot growth in early spring, progressed through rapid vegetative development, and subsequently transitioned to reproductive development. Nutrient availability during this period may therefore influence both the establishment of the vegetative framework and the availability of assimilates and mineral nutrients during floral development. Previous studies on container-grown *Hydrangea paniculata ‘Limelight*’ have shown that fertilizer P concentration affects shoot and root growth, with responses depending on the level of P supply rather than increasing continuously with increasing P availability [5]. These findings highlight the importance of considering plant developmental stage in nutrient management rather than relying solely on fertilizer concentration.

Foliar application was performed at the bud stage because this period represents a developmental transition during which vegetative growth and inflorescence development occur concurrently. Accordingly, foliar application at this stage was designed as a supplementary nutrient treatment rather than a replacement for basal fertilization. Previous studies have shown that foliar nutrient application can alter the nutritional status and physiological responses of ornamental plants, although the magnitude of these responses depends on nutrient concentration, leaf condition, developmental stage, and environmental conditions [10,11]. Therefore, KH_2_PO_4_ was applied foliarly at the bud stage in this study to investigate whether supplementary nutrient supply during reproductive development was associated with changes in flowering traits, physiological characteristics, and nutrient allocation.

Monopotassium phosphate (KH_2_PO_4_) provides readily available P and K and is characterized by high water solubility and chemical stability. It can be applied either as basal substrate amendment or as a foliar spray. Studies in ornamental and horticultural crops, including roses, camellias and strawberries, have shown that appropriate KH_2_PO_4_ application rates can improve photosynthetic efficiency, promote nutrient accumulation in floral organs, and extend the flowering period [12,13]. However, the effects of different application methods may vary with application rate, plant species, and developmental stage, and findings from other crops require further validation in *Hydrangea paniculata ‘Limelight’*. Basal application to the substrate provides a route for nutrient uptake by the roots, whereas foliar application can serve as a supplementary means of nutrient supply. Comparing combinations of these two application methods can therefore help elucidate the effects of additional foliar nutrient supplementation under different basal fertilization regimes. Accordingly, this study evaluated four basal KH_2_PO_4_ application rates, with or without foliar application of 0.1% KH_2_PO_4_ at the bud stage, to compare their individual and combined effects on plant growth and physiological performance.

Transcriptome analysis provides insights into plant molecular responses to different nutrient treatments at the gene-expression level, offering complementary evidence for interpreting physiological and phenotypic differences. To date, omics studies in *Hydrangea* have primarily focused on flower color regulation under aluminum stress and responses to low-temperature stress [14,15], whereas studies investigating nutrient responses and associated gene-expression changes in *Hydrangea paniculata* under combined P and K supply remain relatively limited. Studies have highlighted the roles of the PHR-SPX phosphate-signaling pathway in phosphorus homeostasis and the CBL-CIPK network in potassium transport, plant growth, and stress responses [16,17]. These pathways may provide complementary regulatory links between nutrient status and plant physiological responses, although their direct interactions and coordinated effects on carbon metabolism, antioxidant defense, and flowering require further investigation. However, how these regulatory networks respond to nutrient treatments in *Hydrangea paniculata ‘Limelight’*, and how their responses relate to changes in plant growth, photosynthetic physiology, and nutrient allocation, remain poorly understood. Integrating transcriptome sequencing with quantitative reverse transcription PCR (qRT-PCR) can help identify candidate genes associated with different fertilization treatments and characterize their expression patterns. Nevertheless, gene-expression associations alone are insufficient to establish the functional roles of the implicated pathways or demonstrate causal mechanisms.

In this study, one-year-old potted cuttings of *Hydrangea paniculata ‘Limelight’* were used to investigate the effects of four basal KH_2_PO_4_ foliar applications at the bud stage, in a two-factor experimental design. An untreated control and a nitrogen-only control were also included. Plant vegetative growth, root architecture, photosynthetic pigment content, gas exchange parameters, antioxidant responses, and the accumulation and allocation of N, P, and K among plant organs were systematically evaluated. Principal component analysis (PCA) was performed to integrate the multiple measured traits into a comprehensive assessment. In addition, transcriptome analysis was conducted on representative treatments, and the expression patterns of selected differentially expressed genes (DEGs) were validated by quantitative reverse transcription PCR (qRT-PCR). The specific objectives were to: ① evaluate the effects of basal KH_2_PO_4_ application rate, foliar supplementation, and their interaction on plant growth, stress tolerance and flowering quality of *Hydrangea paniculata ‘Limelight’*; ② characterize N, P, and K accumulation and partitioning among plant organs under different treatments; ③ identify DEGs associated with different P and K supply treatments and explore their potential functions to provide transcript-level insights into the underlying physiological responses; and ④ identify the KH_2_PO_4_ application regime with the best overall performance under the experimental conditions, thereby providing a reference for optimizing fertilization practices in potted *Hydrangea paniculata ‘Limelight’* production.

## 2. Results

### 2.1. Effects of Different KH_2_PO_4_ Concentrations and Fertilization Methods on Growth, Biomass, and Root Traits of Hydrangea paniculata ‘Limelight’

**Figure 1:**
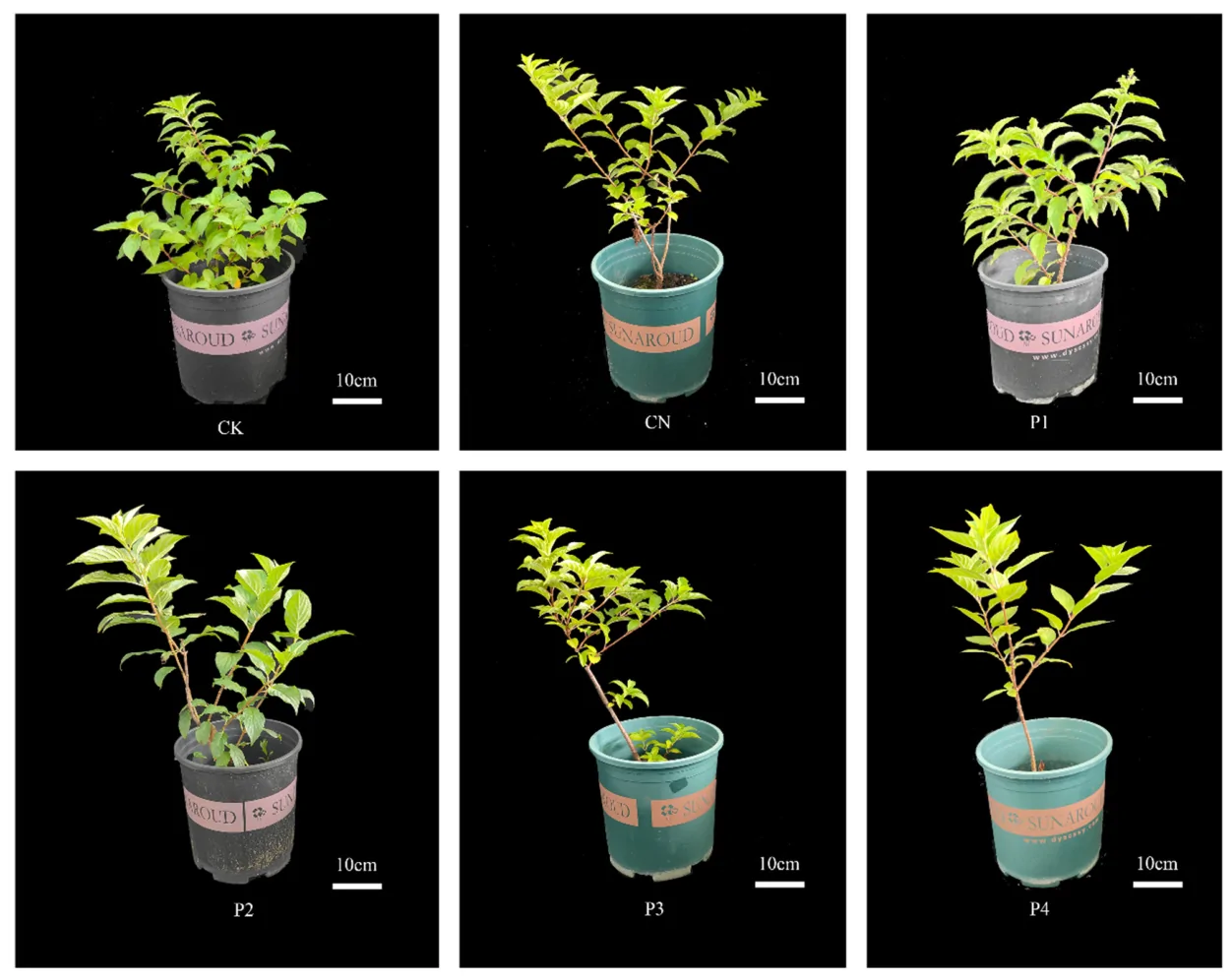
Morphological comparison of *Hydrangea paniculata ‘Limelight’* under different basal KH_2_PO_4_ treatments at 42 days after basal fertilization.

The growth phenotypes of *Hydrangea paniculata ‘Limelight’* were observed and statistically analyzed (Table 1). The sampling time for determination was on the 42nd day after the application of base fertilizer, and topdressing during the budding stage had not yet been carried out. Therefore, Table 1 only reflects the impact of different gradients of KH_2_PO_4_ single base fertilizer on the nutritional growth of *Hydrangea paniculata ‘Limelight’*. Vegetative growth displayed an obvious dose-dependent response to graded P supply. Plants under low-P treatments (CK, CN, P1) produced more branches but showed restrained vertical growth. Moderate basal P (P2, P3) promoted plant height, while the highest P level (P4) significantly thickened stems yet suppressed lateral crown expansion. Under basal fertilization without foliar supplement, P2 generated the longest shoots. Stem diameter of P4 was 24.4% higher than CK, whereas its crown width decreased by 22% relative to CK, indicating that excessive KH_2_PO_4_ restricted horizontal vegetative development (Table 1, Figure 1).

Biomass is a direct indicator of material accumulation during plant growth, and the proportional distribution of biomass among roots, stems, and leaves reflects nutrient utilization patterns (Table 2). Biomass accumulation was significantly affected by basal rate, foliar application, and their interaction for several organs; however, root-to-shoot ratio did not differ significantly among treatments (Table 2).

For treatments receiving only basal substrate fertilization without foliar spray, total biomass continuously decreased with rising basal phosphorus concentration before a slight recovery under the highest P level. CK exhibited the highest total biomass among all treatments without foliar application, although differences among P1–P4 were not statistically significant. Total biomass tended to decrease in the P- and K-deficient CN treatment and the low-P P1 treatment, reaching its lowest levels under P2 and P3. Although total biomass increased markedly under the high-P P4 treatment relative to P2 and P3, it remained lower than that under CK.

When bud emergence stage foliar KH_2_PO_4_ topdressing was supplemented on the basis of basal fertilization, the 4KF treatment achieved the maximum root, stem, and leaf individual organ dry weight and total plant biomass across all treatments. Its total biomass reached 63.34 g, which was 30.4% higher than the blank CK (48.57 g).

The root-to-shoot ratio reflected distinct assimilate partitioning patterns among treatments. The 2KF group possessed the highest root-to-shoot ratio of 1.56, which was significantly higher than the P-K deficient CN group. This result indicated that moderate basal phosphorus combined with budding foliar spray directed photosynthates to preferentially allocate to root systems and promote underground root development. In contrast, the root-to-shoot ratio of the high-P combined treatment 4KF dropped to 1.13, and more assimilates were translocated to above-ground vegetative organs, including stems and leaves.

**Figure 2:**
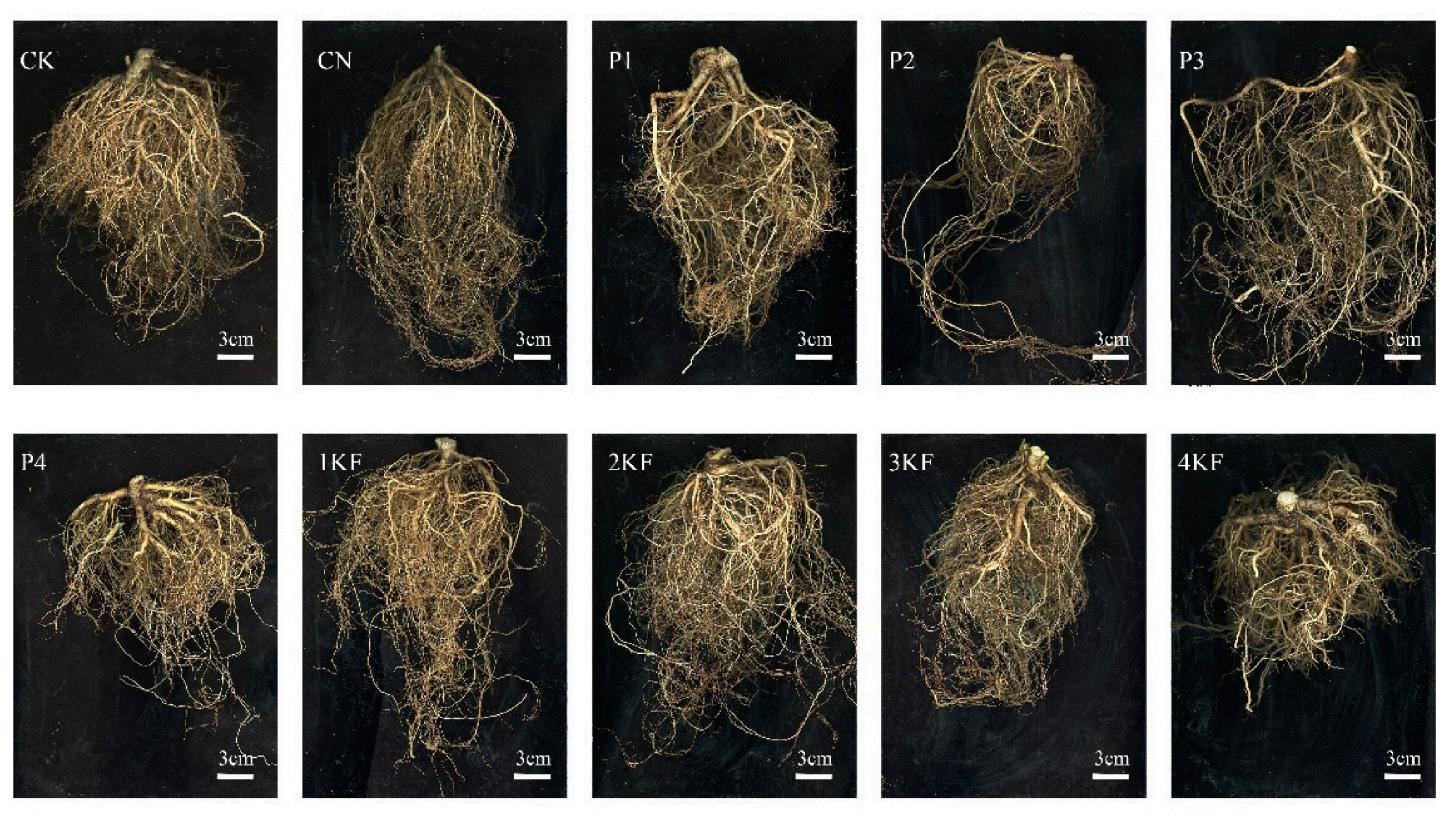
Root morphology of *Hydrangea paniculata ‘Limelight’* plants under different potassium dihydrogen phosphate fertilization treatments.

Root morphology of *Hydrangea paniculata ‘Limelight’* was further analyzed (Table 3; Figure 2). Roots are the primary organs responsible for nutrient acquisition in plants. Two-way ANOVA showed that the basal KH_2_PO_4_ application rate significantly affected total root length, root surface area, and average root diameter, but had no significant effect on root volume. The effects of foliar application and the interaction between basal application rate and foliar application were significant only for total root length and average root diameter (Table 3).

For treatments receiving only basal fertilization, root surface area and root volume of low-nutrient CK and CN groups were markedly higher than those of P1–P4 graded phosphorus treatments, which reflected a compensatory root proliferation strategy under nutrient-limited conditions. However, the trend of total root length was reversed: moderate KH_2_PO_4_ treatments P2 and P3 achieved the maximum total root length among all single-basal groups with optimal root elongation, while total root length declined sharply under high phosphorus P4, demonstrating that excessive phosphorus inhibited longitudinal root extension.

Among the foliar application treatments (1KF–4KF), total root length in 4KF reached 25.10 cm, although its mean value was lower than those in 1KF and 3KF. This pattern suggests that foliar KH_2_PO_4_ application was associated with a lower mean total root length under the conditions of this experiment. Variations in root morphology may be related to plant growth status, root-to-shoot biomass allocation, and nutrient availability. However, the underlying physiological mechanisms require further investigation through direct measurements of root nutrient uptake and plant water status.

By combining biomass partitioning data (Table 2) with root morphology parameters (Table 3), the 2KF treatment showed a relatively high root-to-shoot ratio. However, its root morphological characteristics need to be comprehensively assessed based on the individually measured traits. These findings provide a basis for comparing differences in root architecture and biomass allocation among the fertilization treatments.

### 2.2. Effects of Different KH_2_PO_4_ Concentrations and Fertilization Methods on Flowering Traits of Hydrangea paniculata ‘Limelight’

**Figure 3:**
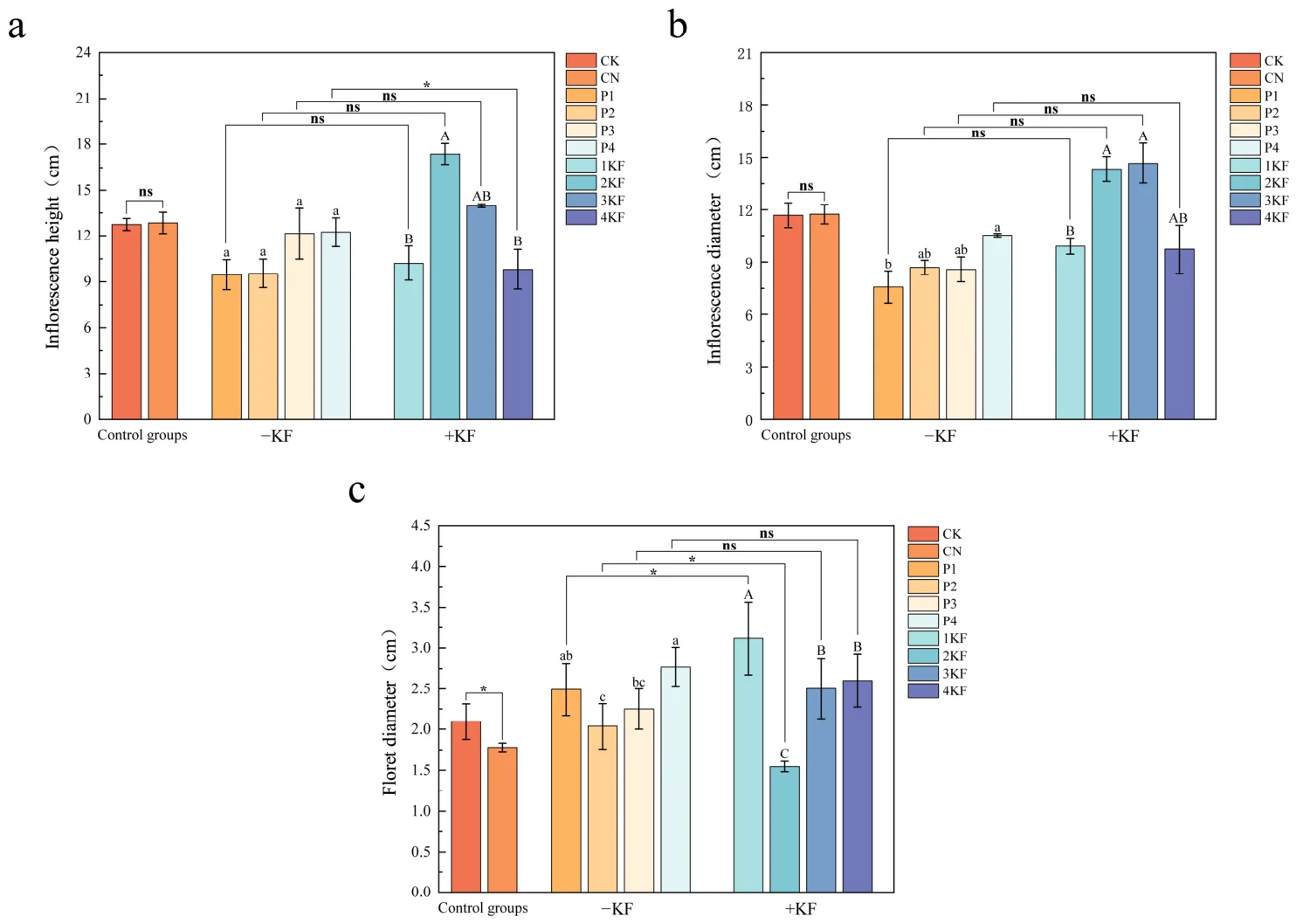
The effect of KH_2_PO_4_ fertilization on the morphology of the flowering part of plants during their peak flowering period. (**a**) Inflorescence height; (**b**) Inflorescence diameter; (**c**) Floret diameter. Lowercase letters indicate significant differences among P1–P4 (no foliar spray). Uppercase letters indicate significant differences among 1KF–4KF (with foliar spray). Asterisks indicate significant differences between -KF and +KF within the same basal P level (* *p* < 0.05, ** *p* < 0.01, *** *p* < 0.001). ns indicates no significant difference (*p* > 0.05). Data are mean ± SD.

For inflorescence height (Figure 3a), among the treatments without foliar application (P1–P4), P3 and P4 exhibited greater inflorescence heights than P1 and P2. Among the foliar application treatments (1KF–4KF), 2KF had the greatest inflorescence height, which was significantly higher than those of 1KF and 4KF.

For inflorescence diameter (Figure 3b), P4 had the largest diameter under non-foliar application conditions, significantly exceeding that of P1. Under foliar application conditions, 3KF exhibited the largest inflorescence diameter, although it did not differ significantly from 2KF; however, it was significantly greater than that of 1KF. These results suggest that foliar KH_2_PO_4_ application combined with intermediate basal application rates (2KF and 3KF) may promote inflorescence expansion relative to the corresponding basal-only treatments (P2 and P3). In particular, 2KF performed well in terms of both inflorescence height and diameter, suggesting that this treatment may favor the development of larger inflorescences under the conditions of this experiment.

For individual floret diameter (Figure 3c), P4 had the largest florets among the non-foliar treatments, whereas P2 had the smallest. Among the foliar application treatments, 1KF exhibited the largest individual floret diameter, significantly exceeding those of the other foliar application treatments. Thus, although 2KF produced relatively large inflorescences in terms of height and diameter, its individual florets were comparatively small. This combination suggests a potential difference between overall inflorescence size and individual floret size; however, further measurements of inflorescence density and overall ornamental quality would be required to determine its implications for visual appeal.

As shown by the flowering phenology data in Table 4, first flowering occurred from late June to mid-to-late July across treatments. Treatment 3KF had the earliest onset of flowering, whereas 4KF had the latest. Total flowering duration also varied among treatments. The longest flowering period was observed in 3KF (91 d), followed by 2KF (90 d), whereas CK had the shortest flowering period (73 d). Relative to CN, flowering duration was extended by 13 d in 2KF, 14 d in 3KF, and 12 d in 4KF. Treatment P3 also extended flowering duration by 9 d compared with CN. Considering inflorescence morphology and flowering duration together, 2KF combined relatively high inflorescence height and diameter with a flowering period of 90 d. Treatment 3KF had a slightly longer flowering period (91 d), with inflorescence dimensions comparable to those of 2KF. Although 4KF extended flowering duration by 12 d relative to CN, it delayed flowering onset and exhibited a comparatively smaller inflorescence diameter.

**Figure 4:**
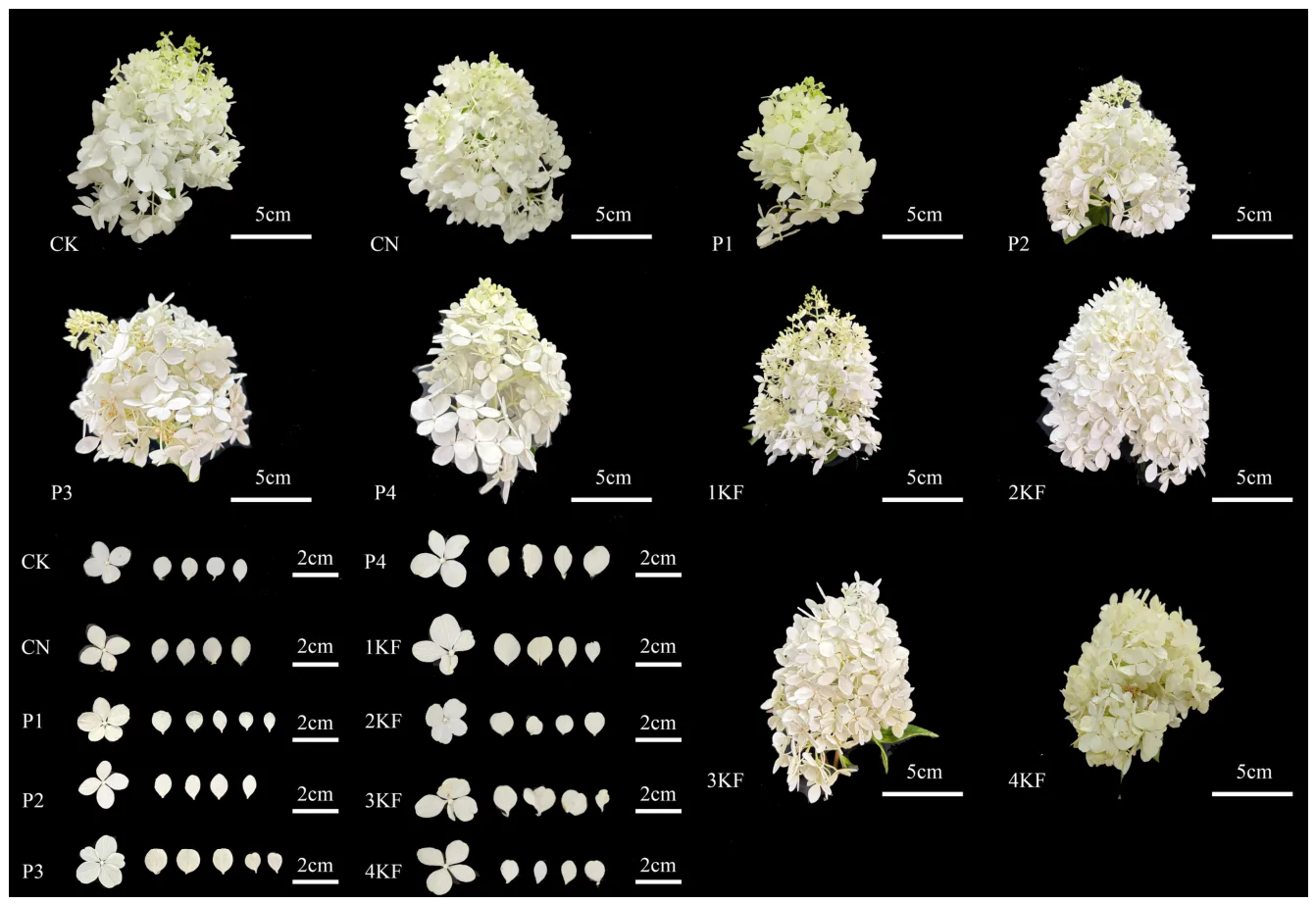
Morphology of the flower part of the plant during its peak flowering period after KH_2_PO_4_ fertilization.

The morphological differences of inflorescences among different fertilization treatments can be visually observed in Figure 4.

Overall, the effects of different KH_2_PO_4_ fertilization regimes on flowering phenology and inflorescence traits were not uniform. Within the range of treatments tested, 2KF performed well in terms of inflorescence height and diameter, whereas 1KF produced the largest individual florets. The relative advantages of each treatment therefore depended on the ornamental trait considered and should be evaluated alongside flowering duration, inflorescence number, and other relevant indicators.

### 2.3. Effects of Different Fertilization Treatments on Photosynthetic Characteristics of Hydrangea paniculata ‘Limelight’

Two-way ANOVA showed that the basal KH_2_PO_4_ application rate significantly affected leaf photosynthetic pigment contents, net photosynthetic rate (A), and the maximum quantum efficiency of photosystem II (PSII), as indicated by Fv/Fm (*p* < 0.05; Table 5, Table 6 and Table 7). Foliar KH_2_PO_4_ application at the visible bud stage significantly affected leaf pigment contents and net photosynthetic rate (*p* < 0.05), but had no significant main effect on chlorophyll fluorescence parameters. The interaction between basal application rate and foliar application significantly affected photosynthetic pigment content and Fv/Fm (*p* < 0.05). In contrast, transpiration rate (E), intercellular CO_2_ concentration (Ci), stomatal conductance (Gs), initial fluorescence (Fo), maximal fluorescence (Fm) and the potential activity of PSII (Fv/Fo) were not significantly affected by either foliar application or their interaction between the two factors (*p* > 0.05).

Among the treatments without foliar application (P1–P4), P1 exhibited significantly higher contents of chlorophyll a (0.77 mg g^−1^), chlorophyll b (0.34 mg g^−1^), total chlorophyll (1.11 mg g^−1^), and carotenoids (2.48 mg g^−1^) than P2, P3, and P4 (Table 5). Pigment contents were relatively low at the intermediate KH_2_PO_4_ application rates in P2 and P3, whereas a slight increase was observed in P4; however, pigment contents in P4 remained lower than those in P1. The high pigment contents under the low KH_2_PO_4_ application rate may represent a compensatory response that helps maintain light-harvesting capacity under limited P and K availability. Conversely, the lower pigment contents under intermediate application rates may be associated with changes in resource allocation when nutrient availability is more favorable.

Net photosynthetic rate (A) followed a unimodal pattern across the basal-only treatments, reaching a maximum of 2.84 μmol m^−2^ s^−1^in P3, significantly higher than the values in P1 and P2 (Table 6). This result suggests that an appropriate basal KH_2_PO_4_ application rate may support greater carbon assimilation. Although pigment contents were higher in P1, this did not translate into a higher net photosynthetic rate, indicating that greater pigment accumulation alone may not compensate for the limitations imposed by an inadequate basal nutrient supply. For chlorophyll fluorescence, differences among treatments were not statistically significant. Nevertheless, P2 and P3 showed numerically higher Fv/Fm values than P1 and P4, suggesting a possible advantage of intermediate KH_2_PO_4_ application rates in maintaining PSII photochemical efficiency, although this trend should be interpreted cautiously given the lack of statistical significance (Table 7).

Among the foliar application treatments (1KF–4KF), 4KF exhibited significantly higher leaf photosynthetic pigment contents than the other treatments, with higher values than those in the corresponding basal-only treatment, P4 (Table 5). For gas exchange, 3KF had the highest net photosynthetic rate (2.85 μmol m^−2^ s^−1^; Table 6). Regarding chlorophyll fluorescence, 2KF exhibited the highest Fv/Fm value among all treatments (0.76), significantly exceeding those of 1KF and 4KF (Table 7). These results indicate that foliar KH_2_PO_4_ application did not uniformly improve all aspects of photosynthetic performance. Instead, the treatments showed different response patterns in pigment accumulation, carbon assimilation, and photochemical efficiency: 4KF was associated with greater pigment accumulation, 3KF with a higher net photosynthetic rate, and 2KF with a higher Fv/Fm value. Overall, each treatment showed a different pattern of photosynthetic responses (Table 5, Table 6 and Table 7), indicating that no single parameter is sufficient to characterize overall photosynthetic performance.

**Figure 5:**
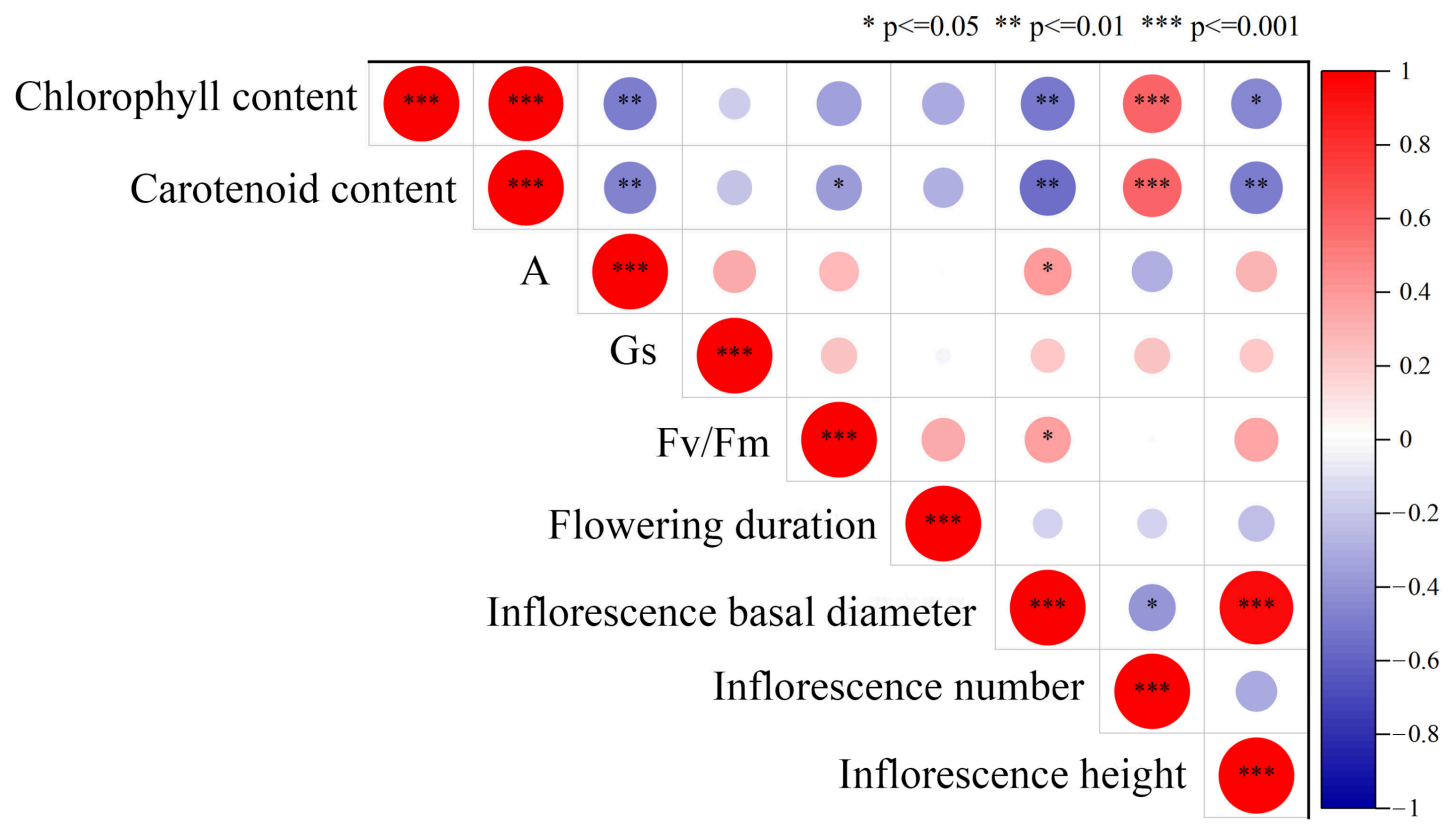
Correlation heatmap of photosynthetic characteristics and flowering quality. Note: * indicates *p* ≤ 0.05, ** indicates *p* ≤ 0.01, *** indicates *p* ≤ 0.001. All indices were determined at full bloom after foliar fertilization.

Photosynthetic products provide the material basis for floral organ development, and the two are intrinsically linked (Figure 5). Correlation analysisrevealed a highly significant positive correlation between chlorophyll and carotenoid contents (*p* ≤ 0.001). Both types of photosynthetic pigments were significantly negatively correlated with the net photosynthetic rate (A) (*p* < 0.01), indicating that higher pigment contents were associated with a lower carbon assimilation rate under the conditions of this experiment. Chlorophyll and carotenoid contents were also positively correlated with inflorescence number but significantly negatively correlated with inflorescence basal diameter and inflorescence height (*p* < 0.05). These relationships suggest that greater pigment accumulation was associated with more inflorescences but smaller inflorescences overall. In contrast, both the net photosynthetic rate (A) and the maximum quantum efficiency of photosystem II (Fv/Fm) were significantly positively correlated with inflorescence basal diameter (*p* ≤ 0.05). Neither A nor Fv/Fm was significantly correlated with flowering duration or inflorescence height.

Overall, these results indicate that photosynthetic pigment accumulation, carbon assimilation capacity, and photochemical efficiency exhibited distinct patterns of association with flowering traits in this experiment. Greater pigment accumulation was associated with a higher inflorescence number but smaller inflorescence dimensions, whereas higher net photosynthetic rates and Fv/Fm values were associated with greater inflorescence basal diameter. These findings suggest that the relationships between photosynthetic characteristics and flowering traits varied depending on the trait examined; however, the correlations do not establish causal relationships.

### 2.4. Effects of Different Fertilization Treatments on Physiological Characteristics of Hydrangea paniculata ‘Limelight’

**Figure 6:**
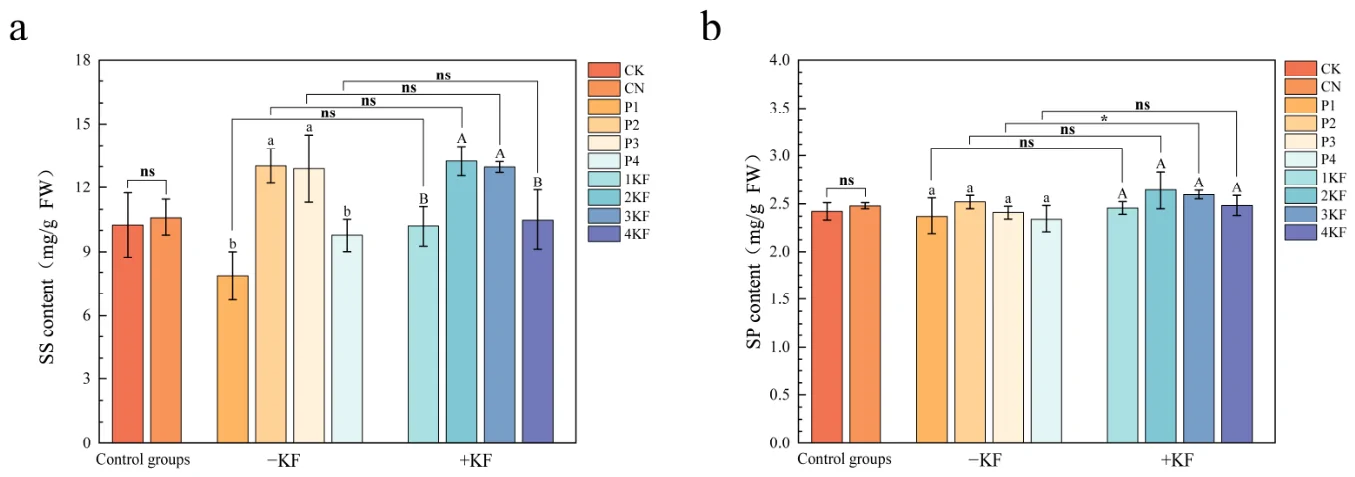
Contents of osmotic adjustment substances in leaves of *Hydrangea paniculata ‘Limelight’* under various KH_2_PO_4_ fertilization treatments. (**a**) Soluble sugar (SS) content; (**b**) Soluble protein (SP) content. Note: Treatment abbreviations are listed in Table 8. CK and CN were analyzed by an independent-samples *t*-test (ns = not significant, *p* > 0.05). Lowercase letters indicate significant differences among P1–P4 (no spray), and uppercase letters indicate significant differences among 1KF–4KF (with spray). Asterisks (*) indicate significant differences between −KF and +KF within the same basal P level. Data are mean ± SD, as shown below.

Osmotic adjustment substances are essential for plants to maintain cellular osmotic pressure, resist environmental stress, and ensure material transport. Phosphorus–potassium fertilization significantly affected soluble sugar (SS) content in the leaves of *Hydrangea paniculata ‘Limelight’* at the full-bloom stage (Figure 6a). Among the treatments without foliar application (P1–P4), soluble sugar contents were highest in P2 and P3, both of which were significantly higher than those in P1 and P4. Among the foliar application treatments (1KF–4KF), 2KF and 3KF exhibited the highest soluble sugar contents, significantly exceeding those of 1KF and 4KF. Treatment 2KF had the highest soluble sugar content, reaching 13.12 mg g^−1^ fresh weight (FW), approximately 29.7% higher than that in the untreated control (CK), followed by P2 and P3. In contrast, P1, which received the lowest basal KH_2_PO_4_ application rate, had the lowest soluble sugar content (7.83 mg g^−1^ FW), significantly lower than those in P2 and P3. Overall, the intermediate basal KH_2_PO_4_ application rates (P2 and P3), both with and without supplementary foliar application (2KF and 3KF), were associated with significantly greater soluble sugar accumulation in leaves.

As shown in Figure 6b, treatment 2KF had the highest soluble protein (SP) content. However, no significant difference was observed between CK and the nitrogen-only control (CN). Similarly, no significant differences in SP content were detected among the treatments without foliar application (P1–P4) or among those with foliar application (1KF–4KF). Overall, SP contents varied only slightly among treatments, suggesting that combined basal and foliar KH_2_PO_4_ application had a limited effect on soluble protein accumulation under the conditions of this experiment. The observed osmotic adjustment responses were more clearly reflected in changes in soluble sugar content.

**Figure 7:**
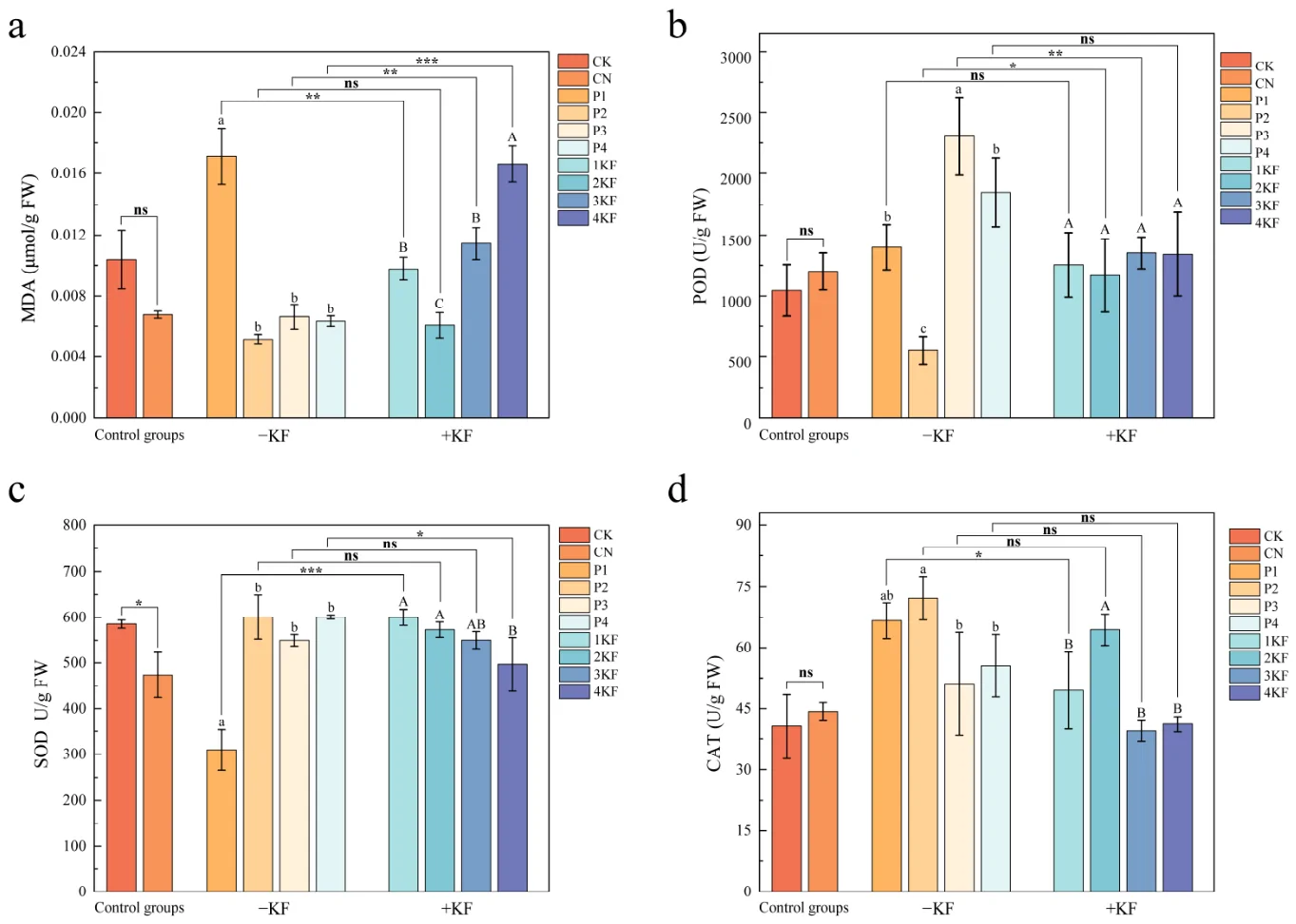
MDA content and antioxidant enzyme activities in leaves of *Hydrangea paniculata ‘Limelight’* under different KH_2_PO_4_ fertilization treatments. Note: (**a**) MDA concentration; (**b**) POD activity; (**c**) total SOD activity; (**d**) CAT activity. Treatment codes are shown in Table 8. CK and CN were analyzed by an independent-samples *t*-test (ns = not significant, *p* > 0.05). Lowercase letters indicate significant differences among P1–P4 (no spray), and uppercase letters indicate significant differences among 1KF–4KF (with spray). Asterisks (*) indicate significant differences between −KF and +KF within the same basal P level. Data are mean ± SD.

Antioxidant enzyme activities and malondialdehyde (MDA) content are commonly used as indicators of oxidative stress and membrane lipid peroxidation in plants. Regarding MDA content (Figure 7a), among the treatments without foliar application, P1 had the highest MDA content, significantly exceeding those of P2, P3, and P4. Treatment P2 had the lowest MDA content (0.005 μmol g^−1^ FW), approximately 70.6% lower than that of P1. Among the foliar application treatments, 2KF exhibited the lowest MDA content, significantly lower than those of 1KF, 3KF, and 4KF. The effects of foliar application on MDA content varied with the basal KH_2_PO_4_ application rate. MDA content decreased significantly in 1KF compared with P1, whereas it increased significantly in 3KF and 4KF compared with P3 and P4, respectively. No significant difference was observed between P2 and 2KF. These results suggest that foliar KH_2_PO_4_ application reduced membrane lipid peroxidation under the lowest basal application rate but was associated with increased MDA accumulation at the intermediate-high and highest rates.

For peroxidase (POD) activity (Figure 7b) among the treatments without foliar application, P3 exhibited the highest activity (2312 U g ^−1^FW), significantly exceeding those of P1, P2, and P4, whereas P2 had the lowest activity. No significant differences were observed among the foliar application treatments (1KF–4KF). Comparisons between treatments with and without foliar application at the same basal KH_2_PO_4_ rate showed that POD activity increased significantly in 2KF relative to P2 but decreased significantly in 4KF relative to P4. No significant differences were detected between P1 and 1KF or between P3 and 3KF. These results indicate that the effect of foliar KH_2_PO_4_ application on POD activity depended on the basal application rate. The relatively low POD activity observed under some treatments may reflect differences in antioxidant responses among nutrient regimes; however, the underlying physiological mechanisms require further investigation.

For superoxide dismutase (SOD) activity (Figure 7c), among the treatments without foliar application, P1 had the lowest activity, significantly lower than those of P2, P3, and P4. No significant differences were observed among P2, P3, and P4. Among the foliar application treatments, 1KF exhibited the highest SOD activity, significantly exceeding that of 4KF. Foliar application significantly increased SOD activity in 1KF relative to P1, whereas no significant differences were observed between P2 and 2KF or between P3 and 3KF. In contrast, SOD activity decreased significantly in 4KF relative to P4. These findings suggest that foliar KH_2_PO_4_ application enhanced SOD activity under the lowest basal application rate, whereas its effects were limited or negative at higher basal application rates.

For catalase (CAT) activity (Figure 7d), among the treatments without foliar application, P2 exhibited the highest activity (75.2 U g^−1^ FW), significantly exceeding those of P3 and P4; however, the difference between P1 and P2 was not significant. Among the foliar application treatments, 2KF had the highest CAT activity (65.8 U g^−1^ FW), significantly exceeding those of 1KF, 3KF, and 4KF. Foliar application significantly decreased CAT activity in 1KF relative to P1, whereas no significant differences were observed between the corresponding treatments at the other basal KH_2_PO_4_ application rates. Thus, foliar KH_2_PO_4_ application did not consistently increase CAT activity, and its effect varied with the basal nutrient supply.

Considering all four indicators together, P2 exhibited relatively low MDA content and POD activity, together with relatively high SOD and CAT activities, under non-foliar application conditions. A broadly similar pattern was observed in 2KF, which had the lowest MDA content and the highest CAT activity among the foliar application treatments, together with relatively low POD activity. These results suggest that the intermediate basal KH_2_PO_4_ application rate, particularly when combined with foliar application (2KF), was associated with lower membrane lipid peroxidation and a distinct antioxidant enzyme activity profile. The relatively low POD activity in these treatments indicates that higher POD activity was not necessarily associated with lower MDA accumulation under the conditions of this experiment.

In contrast, P1 exhibited relatively high MDA content, suggesting a greater degree of membrane lipid peroxidation under the lowest basal KH_2_PO_4_ application rate. Foliar application under this condition was associated with a significant decrease in MDA content and an increase in SOD activity, accompanied by a decrease in CAT activity. At the higher basal application rates, foliar application was associated with increased MDA content, and SOD activity also decreased significantly in 4KF relative to P4. Overall, the effects of foliar KH_2_PO_4_ application on antioxidant responses depended on the basal application rate. Under the lowest basal application rate, foliar application was associated with lower MDA content and higher SOD activity, whereas CAT activity decreased. Although changes in hydrogen peroxide (H_2_O_2_) metabolism may contribute to the observed responses, the proposed mechanisms cannot be established from enzyme activity and MDA measurements alone and require further investigation.

### 2.5. Nitrogen, Phosphorus, and Potassium Contents and Accumulation in Different Organs

**Figure 8:**
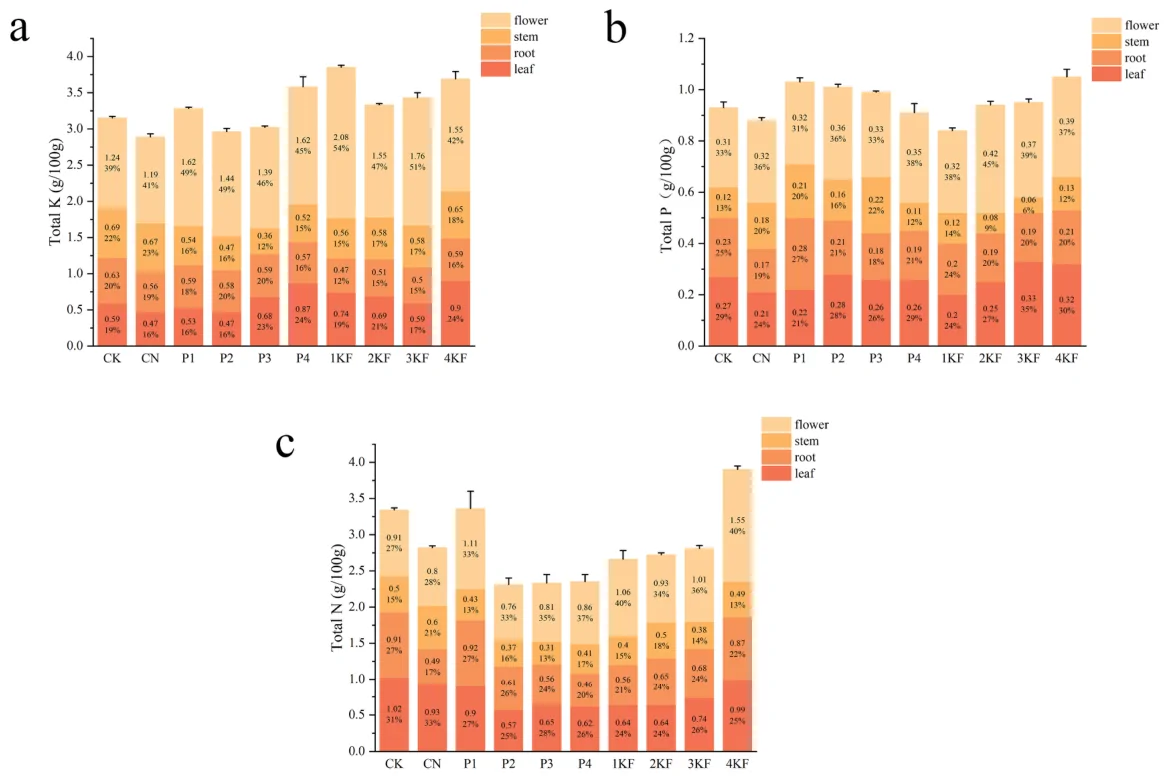
N, P and K accumulation and organ partitioning in *Hydrangea paniculata ‘Limelight’* under different KH_2_PO_4_ treatments at late bloom. Note: (**a**) Total potassium accumulation and partitioning ratio across organs; (**b**) Total phosphorus accumulation and partitioning ratio across organs; (**c**) Total nitrogen accumulation and partitioning ratio across organs. CK: blank control; CN: substrate without additional P and K.

To clarify the partitioning characteristics of nutrients between vegetative and reproductive organs under graded P-K supply, the total accumulation and organ allocation ratio of nitrogen, phosphorus and potassium in leaves, roots, stems and inflorescences were determined and analyzed (Figure 8).

Among the treatments receiving basal substrate fertilization without foliar KH_2_PO_4_ application at the visible bud stage, the nitrogen-only control (CN) exhibited the lowest whole-plant accumulation of N, P, and K. With the increase in basal KH_2_PO_4_ concentration, whole-plant P accumulation gradually decreased, while N accumulation declined under moderate- and high-P treatments P2, P3 and P4, indicating excessive substrate phosphorus supply inhibited plant N uptake to a certain extent. Total K accumulation continuously increased along rising basal phosphorus gradients, and basal KH_2_PO_4_ application significantly promoted plant K accumulation. In terms of organ partitioning, nutrients were mostly retained in vegetative organs (leaves and roots) under single basal fertilization, and the allocation proportion of N, P and K in inflorescences remained relatively low.

Compared with the corresponding basal-only treatments, foliar KH_2_PO_4_ application at the visible bud stage significantly increased whole-plant N, P, and K accumulation at each basal KH_2_PO_4_ application rate. These results suggest that foliar P and K supplementation enhanced whole-plant nutrient accumulation when combined with basal fertilization. The three nutrients exhibited a common pattern of organ allocation: all foliar application treatments increased the proportions of N, P, and K allocated to the inflorescences, indicating greater nutrient partitioning to reproductive organs. Among the treatments, 2KF, which combined an intermediate basal KH_2_PO_4_ application rate with foliar supplementation, showed the highest proportion of P allocated to the inflorescences across all treatments. Treatment 1KF had the highest whole-plant K accumulation, with 54% of total K allocated to the inflorescences. In contrast, 4KF, which received the highest basal KH_2_PO_4_ application rate along with foliar supplementation, exhibited the highest whole-plant N accumulation, with 40% of total N allocated to the inflorescences.

**Figure 9:**
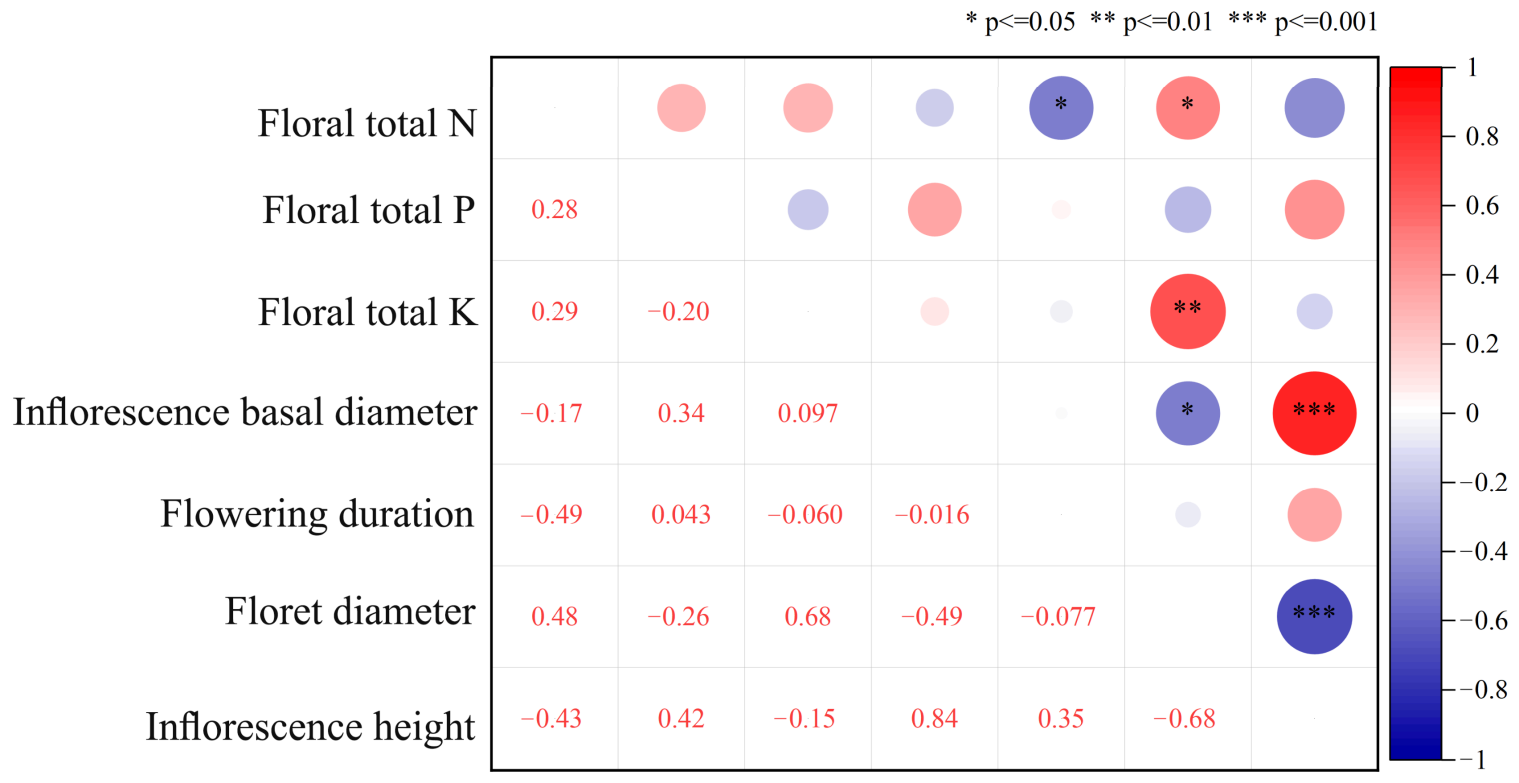
Correlation analysis between nutrient contents in floral organs and flowering quality indicators.

Significant correlations existed between floral nutrient concentrations and flowering traits (Figure 9). Overall, inflorescence traits were more strongly associated with N and K contents in the floral organs, whereas P content showed weaker associations with the measured floral traits. Floral-organ N content was significantly positively correlated with inflorescence basal diameter and significantly negatively correlated with flowering duration (*p* < 0.05). Floral-organ K content was very significantly positively correlated with inflorescence basal diameter (*p* < 0.01). In addition, inflorescence basal diameter was significantly negatively correlated with individual floret diameter but significantly positively correlated with inflorescence height. Individual floret diameter was also significantly negatively correlated with inflorescence height.

These correlation patterns suggest that the allocation of N and K within floral organs may be associated with variation in the ornamental traits of *Hydrangea paniculata ‘Limelight’*. Greater nutrient allocation to the inflorescence framework may be associated with larger inflorescences and greater inflorescence height, potentially accompanied by smaller individual florets. Conversely, greater allocation to individual florets may be associated with increased floret size but smaller overall inflorescence dimensions and a potentially shorter flowering period. These interpretations remain hypothetical, as the observed correlations do not establish the direction of nutrient transport or a causal relationship between nutrient allocation and floral development.

These findings are broadly consistent with the observed patterns in photosynthetic physiology, antioxidant responses, and flowering traits, and suggest a potential trade-off between overall inflorescence size and individual floret size in relation to internal nutrient allocation. The correlation results therefore provide a basis for further investigation into the relationships between floral nutrient status and ornamental traits. However, conclusions regarding nutrient transport, competition among plant organs, and the underlying physiological mechanisms require further validation through dynamic nutrient-tracing studies or targeted fertilization experiments.

### 2.6. Multi-Index Principal Component Analysis (PCA)

Principal component analysis (PCA) was performed on plant growth, root traits, flowering characteristics, photosynthetic parameters, and nutrient allocation indicators under different KH_2_PO_4_ treatments. PC1 and PC2 explained 32.1% and 19.2% of the total variation, respectively, accounting for 51.3% cumulatively. Because these two components captured approximately half of the total variation, the PCA ordination should be interpreted as a complementary summary of treatment responses rather than a definitive ranking of overall performance.

**Figure 10:**
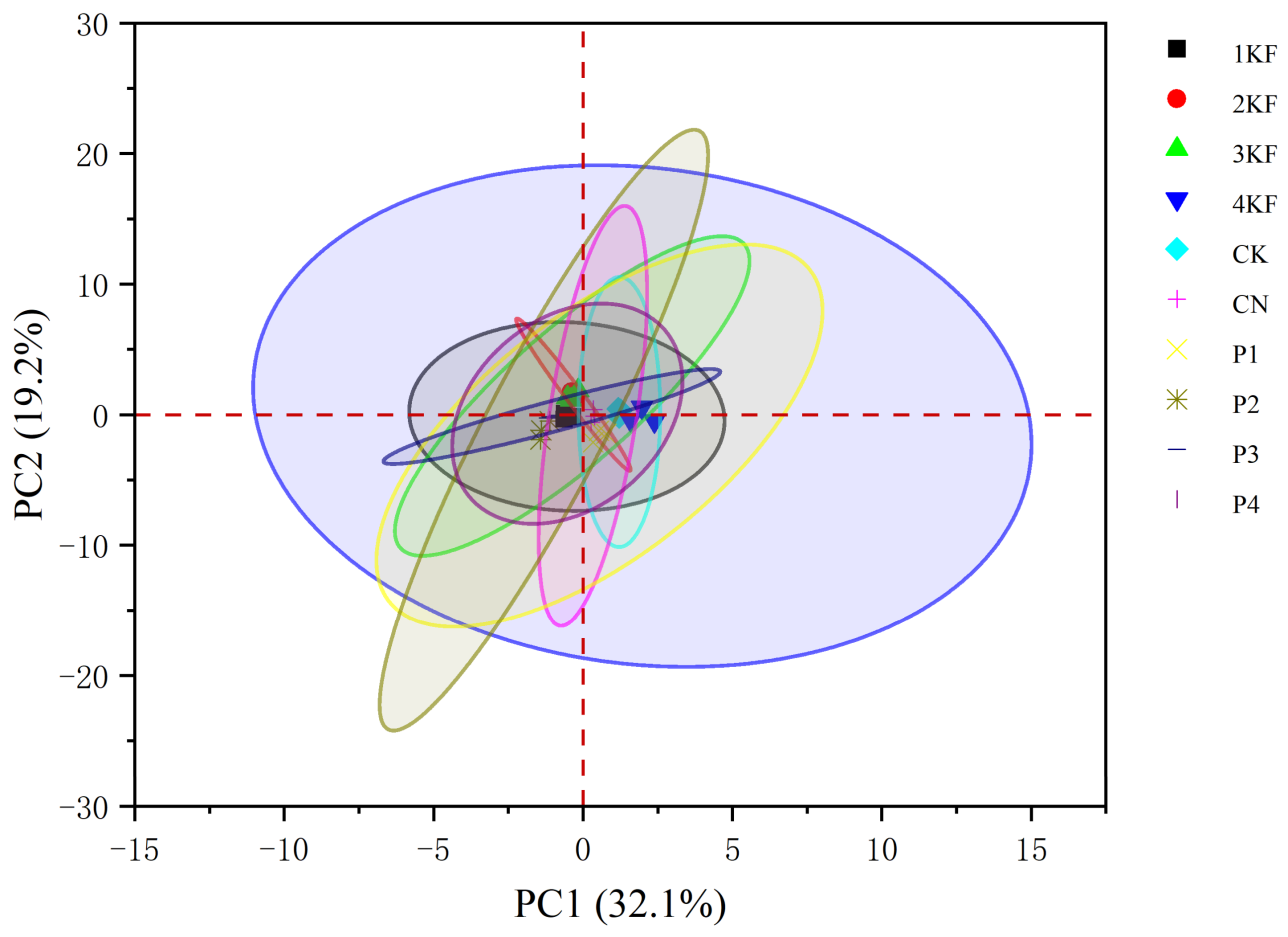
Principal component analysis (PCA) of growth, physiological, nutrient and flowering indices of *Hydrangea paniculata ‘Limelight*’ under all KH_2_PO_4_ fertilization treatments. Note: Ellipses represent 95% confidence intervals. Treatment codes refer to Table 8.

The sample points of the 2KF treatment were the most tightly clustered, and its 95% confidence ellipse was the smallest (Figure 10), indicating that plant traits under this treatment were highly stable and that within-group repeatability was the best. By contrast, the confidence ellipses of treatments such as 4KF, P1, and P2 were larger, indicating greater within-group variation and poorer trait stability. In addition, the 2KF samples did not overlap with CK or CN samples, indicating significant differences in comprehensive performance. This indicates that the samples from treatment 2KF were more tightly clustered in the multivariate space defined by the measured traits, whereas those from some other treatments exhibited greater dispersion.

### 2.7. Transcriptomic Analysis and qRT-PCR Validation

**Figure 11:**
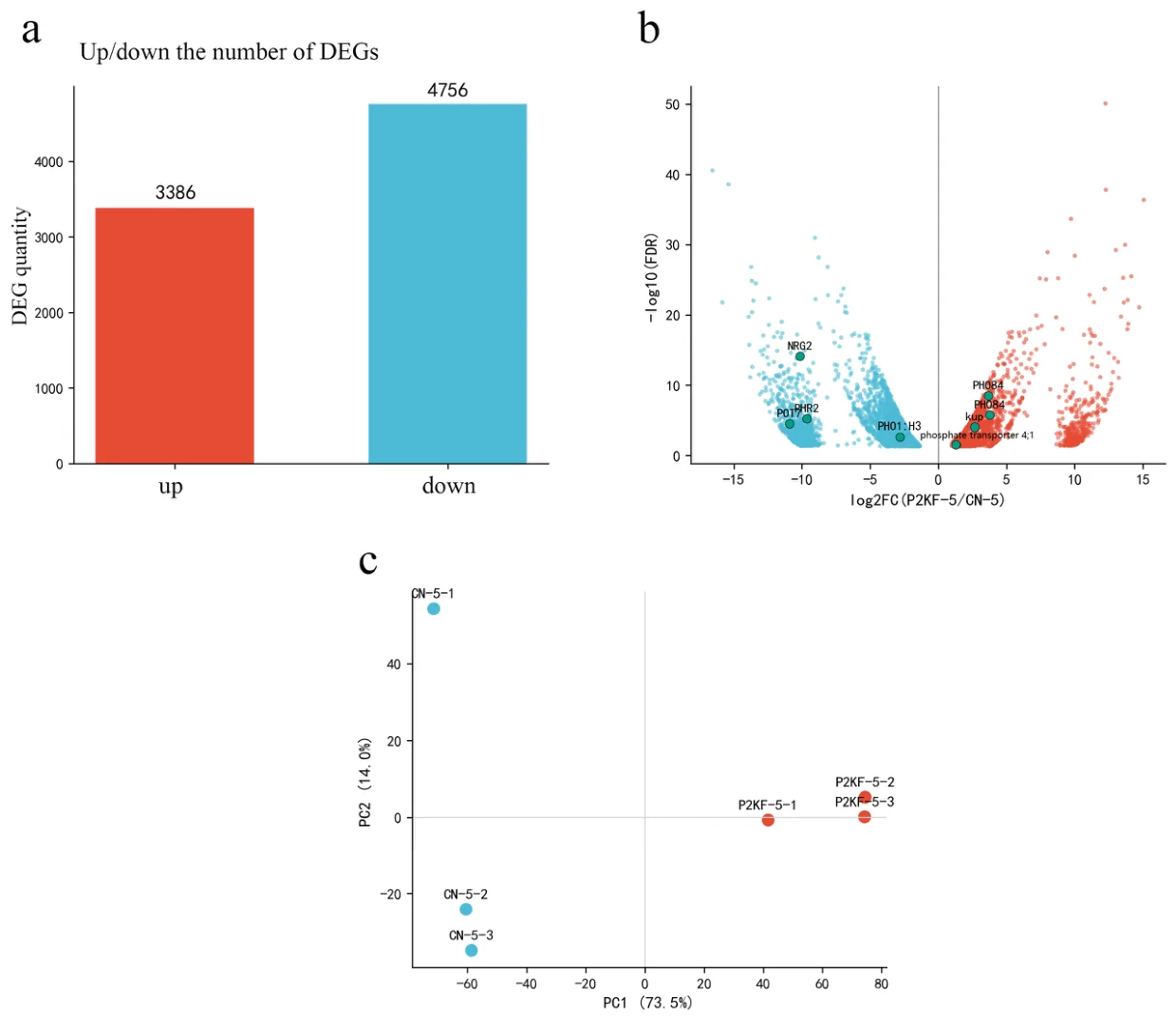
Global comprehensive analysis of differentially expressed genes between the CN and 2KF groups. Note: (**a**) Bar chart of the number of DEGs upregulated and downregulated in the two groups; (**b**) Volcano plot of DEG differences, labeling core nutrient transport genes such as PHT, HAK, and PAP26; (**c**) PCA clustering diagram of the samples. Red represents genes upregulated in 2KF relative to CN, and blue represents genes downregulated; PC1 and PC2 explain 73.5% and 14.0% of the sample expression variations, respectively.

RNA-seq was performed on full-bloom leaf samples of CN (nitrogen-only control without P and K) and 2KF (optimal treatment with 100 mg P_2_O_5_ kg^−1^ basal KH_2_PO_4_ plus budding foliar spray). Q30 values of all libraries were above 93.12%, confirming qualified sequencing data. DEGs were screened under thresholds of |log_2_Fold Change| ≥ 1 and adjusted *p* < 0.05. A total of 8142 DEGs were identified, including 3386 up-regulated and 4756 down-regulated genes (Figure 11a). PCA showed complete separation between CN and 2KF, demonstrating that P-K supply acted as the core factor reshaping leaf transcript profiles (Figure 11c). Key nutrient transporter genes were labeled in the volcano plot: PHT phosphate transporters and HAK potassium transporters were significantly up-regulated in 2KF, while phosphate starvation genes PHR2 and NRG2 accumulated abundantly in CN. These expression patterns suggest that phosphorus starvation signals may be associated with the transcriptional regulation of potassium transport pathways (Figure 11b).

**Figure 12:**
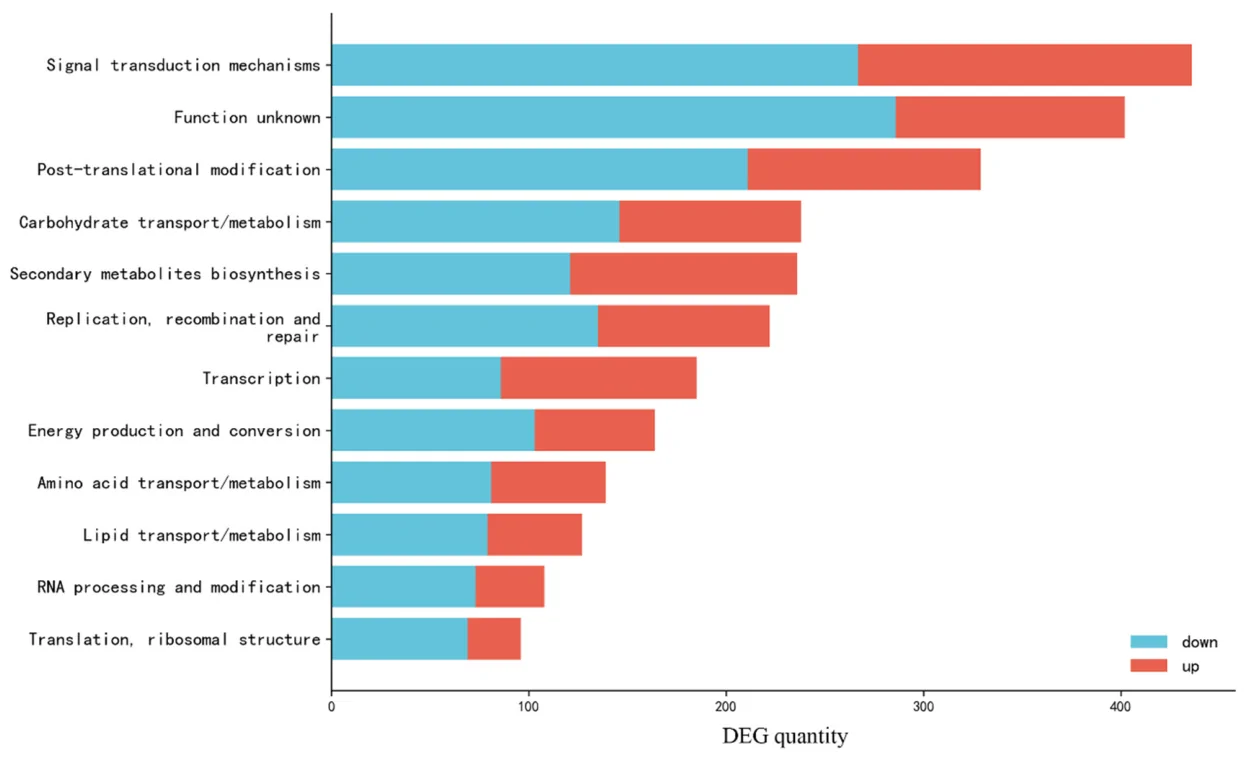
Stacked bar chart of KOG functional classification of all differentially expressed genes. Note: Brick red represents differentially expressed genes upregulated by 2KF, and light blue represents differentially expressed genes downregulated; the vertical axis represents the standard KOG functional entries, and the horizontal axis represents the number of corresponding DEG entries.

KOG classification of all DEGs revealed that signal transduction and carbohydrate transport & metabolism were the two most enriched functional categories (Figure 12). Massive carbon metabolism genes were activated under P-K deficiency to remodel carbon allocation against nutrient limitation; balanced P-K supply alleviated stress signals, down-regulated stress-related carbon transporters and promoted directional translocation of photosynthates.

**Figure 13:**
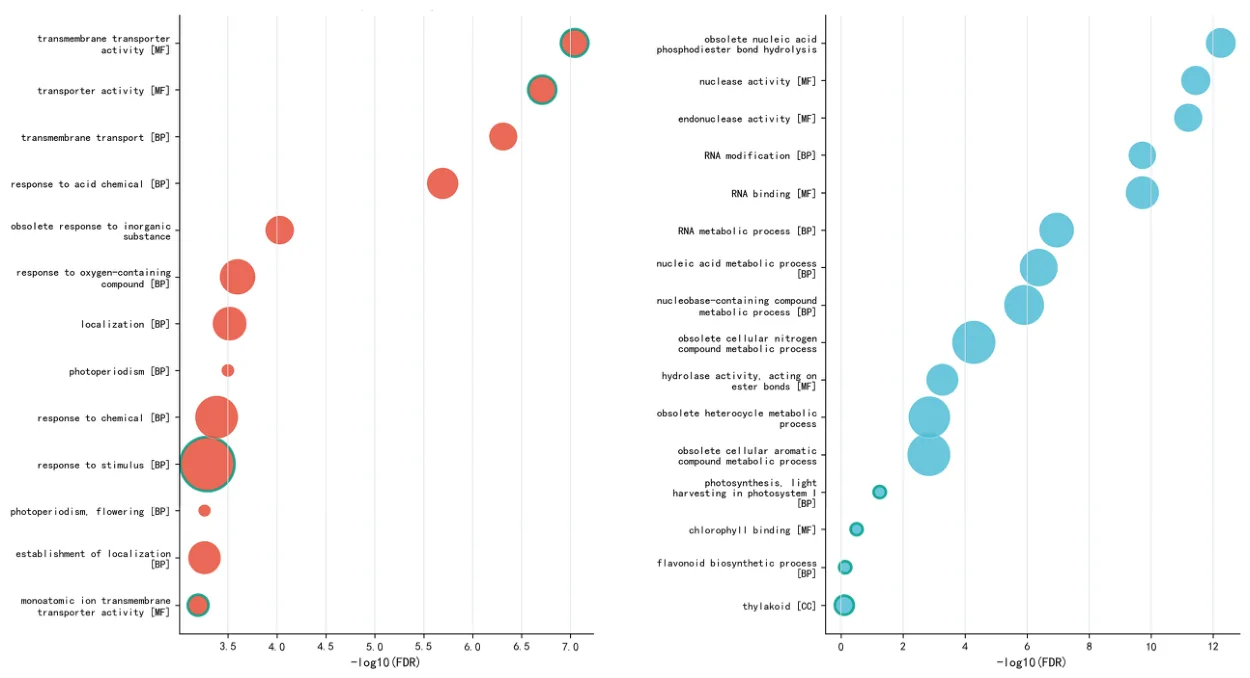
Two-way GO enrichment bubble plot of up- and down-regulated DEGs. Note: Bubble diameter represents the number of differentially regulated genes enriched in that GO entry; larger bubbles indicate more annotated genes. The horizontal axis is −log_10_(FDR), with higher values indicating stronger statistical significance of enrichment. Green borders mark functional entries directly related to phosphorus and potassium absorption, photosynthetic system, flowering regulation, and antioxidant physiology, based on biological function and not bubble size. Red areas represent upregulated genes enriched in 2KF compared to CN, and blue areas represent downregulated genes enriched in 2KF compared to CN.

**Figure 14:**
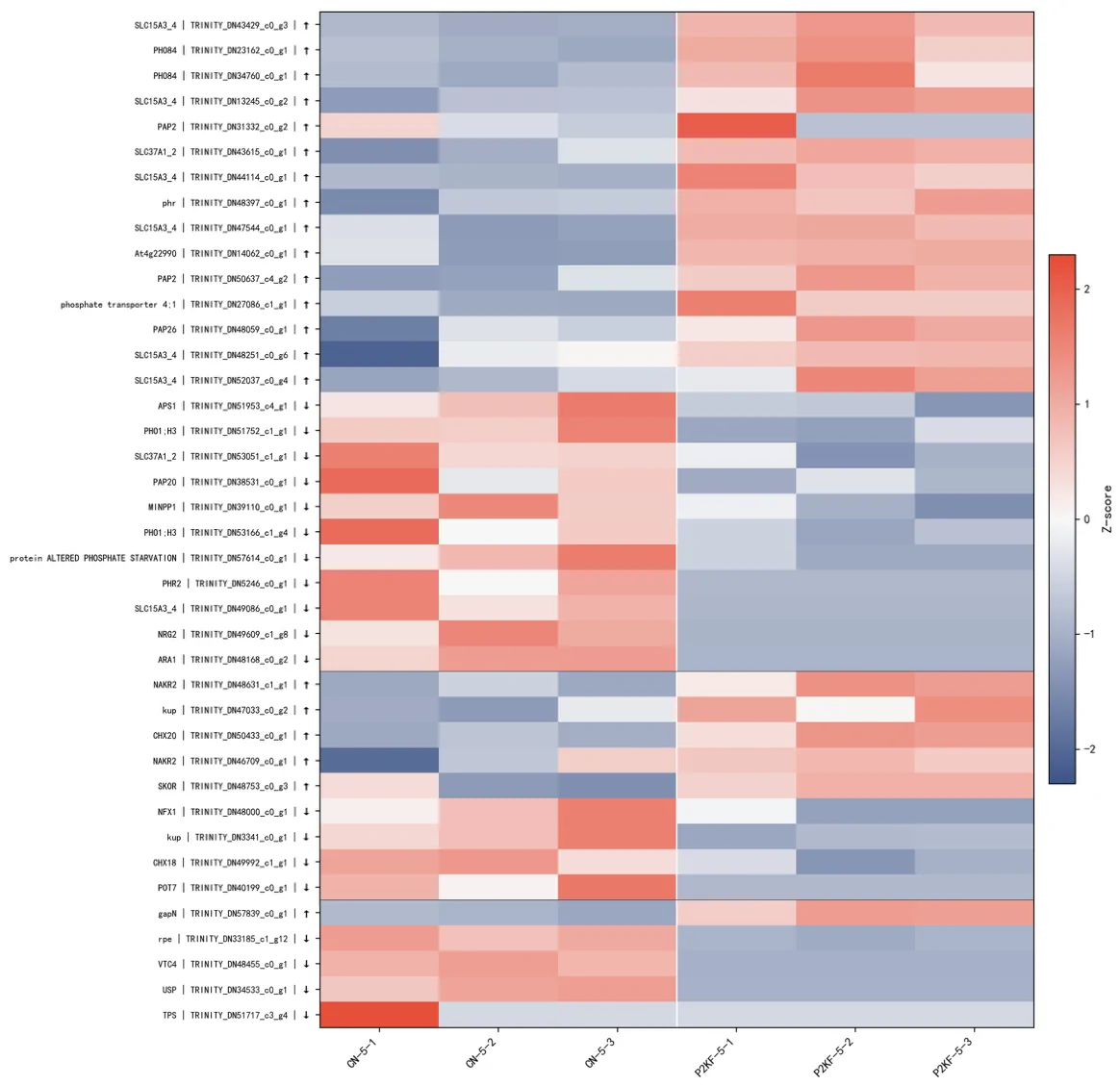
Heatmap of standardized Z-scores for candidate genes for phosphorus and potassium transport and phosphorus metabolism. Note: Color gradient represents gene standardized expression level, red for high expression and blue for low expression; rows represent target nutrient genes and columns represent biological replicates.

Bidirectional GO enrichment illustrated that up-regulated DEGs in 2KF were extremely significantly enriched in ion transmembrane transport, photoperiodic flowering, antioxidant defense and photosynthetic carbon fixation (FDR < 0.001). Genes highly expressed in CN were mainly enriched in phosphate starvation and stress response pathways (Figure 13). The heatmap of core genes involved in P/K transport and phosphorus metabolism further showed that 2KF activated both PHT and HAK nutrient transporters, while phosphate starvation repressors accumulated largely in CN with lower potassium channel expression (Figure 14). These findings provide transcriptional evidence for the coordinated expression of P and K transporter genes under KH_2_PO_4_ application.

**Figure 15:**
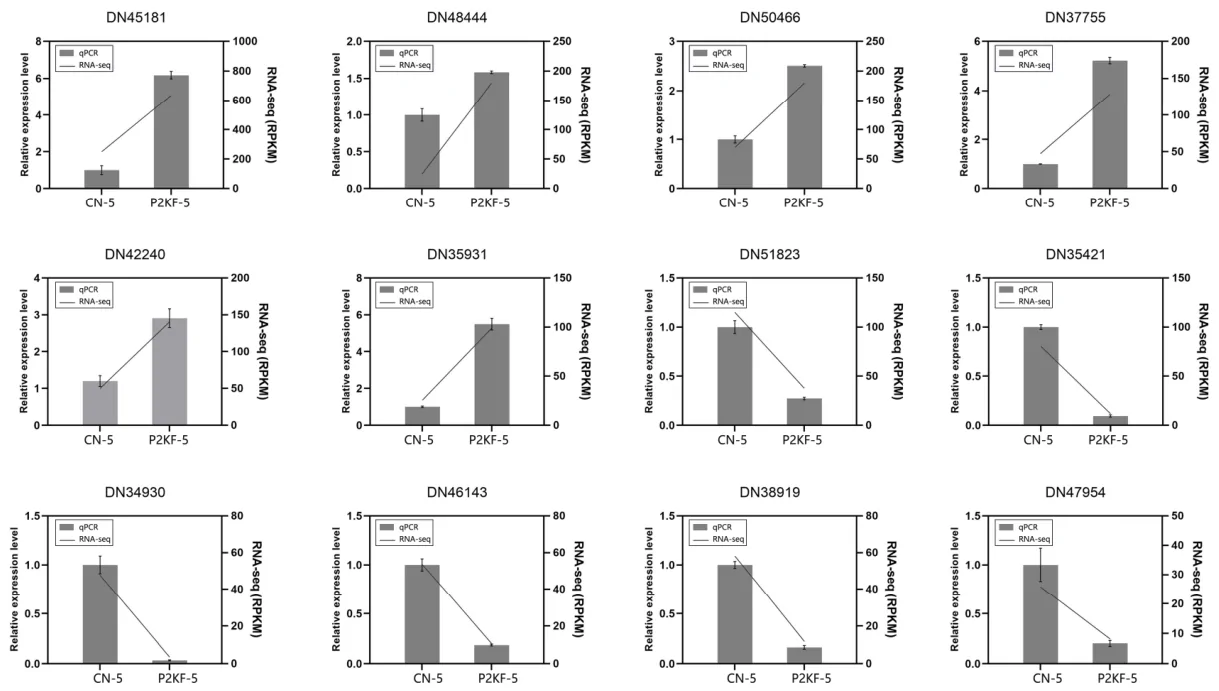
qRT-PCR validation results of 12 key differentially expressed genes under CN and 2KF treatments. Note: The bar chart represents the relative gene expression levels detected by qRT-PCR, and the line represents the gene expression levels obtained by transcriptome sequencing. The gene expression trends are consistent under the two detection methods.

To verify the reliability of the transcriptome sequencing results, this study randomly selected 12 core differentially expressed genes involved in nutrient transport, carbon metabolism, and stress regulation for qRT-PCR quantitative verification (Figure 15). The results showed that the upregulation and downregulation trends of the relative expression abundance of each gene by qRT-PCR were generally consistent with the RPKM expression patterns obtained from RNA-seq sequencing, supporting the reliability of this transcriptome dataset. Functional analysis indicated that the upregulated genes in the 2KF group were mainly involved in starch degradation, carbon and nitrogen metabolism, chloroplast assembly, and antioxidant defense processes, which may be associated with the higher photosynthetic efficiency and stronger stress resistance of plants under this treatment at the molecular level. The significantly downregulated genes were mostly enriched in pathways related to membrane lipid peroxidation, leaf senescence, and persistent stress response, providing molecular clues for understanding how KH_2_PO_4_ supply may alleviate oxidative damage and prolong the ornamental flowering period.

## 3. Discussion

### 3.1. Effects of KH_2_PO_4_ Application on Root Growth and Biomass Allocation

Roots serve as the core organ enabling plants to sense soil nutrients and absorb water and mineral elements [18,19]. Previous studies have shown that under low-P conditions, maize and leguminous plants can enhance P acquisition by modifying lateral root development, increasing the number of root tips, and expanding the root surface area available for nutrient uptake. However, changes in total root length have not been consistent across studies [20,21]. In Rosa roxburghii and rose, both insufficient and excessive P supply may inhibit root growth [13,22]. In the present study, *Hydrangea paniculata ‘Limelight’* exhibited differential root responses to varying KH_2_PO_4_ application rates. Under the low KH_2_PO_4_ application rate, P1 did not show a marked increase in total root length, whereas root surface area and volume were relatively high. This pattern may indicate that plants adjusted root morphology to maintain a relatively large nutrient-absorbing interface, consistent with findings that annual crops can modify root architecture to improve P acquisition under P deficiency [20,21]. At intermediate KH_2_PO_4_ application rates, P2 and P3 exhibited greater total root length, whereas their root surface area and volume were lower than those in P1. This indicates that different root morphological traits did not respond synchronously to KH_2_PO_4_ supply, consistent with previous reports of nonlinear root morphological responses to P availability in R. roxburghii and rose [13,22]. At the highest application rate, P4 reduced total root length and surface area relative to P2/P3, but root diameter and volume were not consistently reduced, indicating high P mainly inhibited longitudinal root extension rather than all root traits. Whether these responses were also influenced by K availability requires further investigation.

Plant biomass, root-to-shoot ratio, biomass allocation, and nutrient accumulation are closely related. Under limited nutrient availability, plants may allocate a greater proportion of biomass to roots to maintain nutrient acquisition, whereas adequate P and K supply may help coordinate root development with aboveground growth. K plays important roles in mineral nutrient transport and osmotic regulation [23], and studies of woody plants have also indicated that adequate K supply can promote N and P uptake and influence biomass allocation. Ref. [13] further reported that an appropriate combination of basal KH_2_PO_4_ application and foliar fertilization could improve nutrient uptake and flowering performance in rose. In the present study, all treatments receiving foliar KH_2_PO_4_ at the visible bud stage had higher root-to-shoot ratios than their corresponding basal-only treatments, with 2KF exhibiting the highest value. This suggests that an appropriate combination of basal application and foliar supplementation may help coordinate belowground and aboveground growth.

Plant nutrient uptake, accumulation, and allocation among organs are jointly influenced by organ function, developmental stage, and resource allocation strategies. Reproductive resource allocation may also vary depending on the metrics used to quantify resource investment [24]. Treatment 4KF exhibited the highest whole-plant accumulation of N, P, and K, but a lower proportion of these nutrients was allocated to the inflorescences. This pattern may reflect differences in resource partitioning between vegetative and reproductive growth. However, this interpretation requires further validation using organ-specific biomass, nutrient concentrations, and nutrient accumulation data.

Overall, *Hydrangea paniculata ‘Limelight’* exhibited dose-dependent responses to KH_2_PO_4_ supply, while the addition of foliar KH_2_PO_4_ application at the visible bud stage resulted in distinct responses in root morphology, root-to-shoot ratio, and nutrient accumulation and allocation. Among the treatments, 2KF had the highest root-to-shoot ratio, suggesting a potentially favorable balance in biomass allocation between belowground and aboveground organs under the conditions of this experiment.

### 3.2. Effects of KH_2_PO_4_ Application on Physiological Characteristics and Flowering Performance

Phosphorus plays an essential role in photosynthetic metabolism and energy conversion, whereas potassium is involved in stomatal regulation, enzyme activity, and osmotic adjustment [8]. In the present study, the low-concentration KH_2_PO_4_ treatment (P1) resulted in relatively high chlorophyll and carotenoid contents but lower net photosynthetic rate (Pn) and maximum quantum efficiency of photosystem II (Fv/Fm), indicating that photosynthetic pigment accumulation did not correspond directly to photosynthetic performance. Similar discrepancies among photosynthetic parameters have been reported in studies of phosphorus deficiency in rice [25]. However, because the phosphorus nutritional status of P1 plants was not directly determined, their photosynthetic responses cannot be directly attributed to phosphorus deficiency. In contrast, plants under the intermediate-concentration treatments (P2 and P3) exhibited better photosynthetic performance, whereas photosynthetic parameters declined under the high-concentration treatment (P4). These findings suggest that the response of *Hydrangea paniculata ‘Limelight’* to KH_2_PO_4_ supply may be concentration-dependent and that higher application rates do not necessarily lead to further improvements in photosynthetic performance.

Photosynthetic carbon assimilation and antioxidant capacity jointly contribute to plant growth and flowering performance. Potassium is involved in reactive oxygen species (ROS) regulation and antioxidant defense, while phosphorus supply also influences carbon metabolism and energy conversion [8]. In this study, the nutrient-deficient control (CN) and the low- and high-concentration treatments (P1 and P4) generally exhibited lower soluble sugar contents and antioxidant enzyme activities, together with higher malondialdehyde (MDA) contents. ROS play important roles in plant signaling and stress responses, but their excessive accumulation may cause oxidative damage to cellular components. Antioxidant enzymes contribute to ROS homeostasis and may help mitigate oxidative damage under stress conditions [26]. The 2KF treatment exhibited relatively high catalase (CAT) activity and a low MDA content, indicating a comparatively favorable antioxidant status. Nevertheless, because ROS levels were not directly measured in this study, these indicators alone are insufficient to establish the underlying oxidative response mechanisms.

Differences in physiological characteristics were also accompanied by variations in flowering performance. CN and P1 had more branches/inflorescences but smaller inflorescences and shorter flowering periods than the optimal 2KF treatment. These findings indicate that inflorescence number and the ornamental quality of individual inflorescences did not follow identical response patterns, and a greater number of inflorescences did not necessarily translate into superior overall ornamental performance. Adequate phosphorus supply can support nutrient accumulation and reproductive development, while potassium contributes to photosynthate transport and reproductive organ development [8,27]. However, the present study does not establish a direct causal relationship between the observed physiological changes and inflorescence traits.

Nutrient accumulation and partitioning among plant organs further highlighted differences among treatments. The 4KF treatment resulted in relatively high whole-plant accumulation of nitrogen, phosphorus, and potassium, but a lower proportion of these nutrients was allocated to the inflorescences. This suggests that greater whole-plant nutrient accumulation does not necessarily correspond to a higher proportion of nutrients allocated to reproductive organs. In contrast, foliar-sprayed treatments generally showed higher proportions of nitrogen, phosphorus, and potassium allocated to the inflorescences, with 2KF exhibiting the highest phosphorus allocation proportion. These findings suggest that nutrient supply levels and fertilization methods may jointly influence nutrient accumulation and partitioning among plant organs, and that whole-plant nutrient accumulation and the relative allocation of nutrients to reproductive organs may respond differently. It should be noted that nutrient allocation proportion is a relative measure; changes in this proportion alone cannot establish whether the absolute amount of nutrients accumulated in the inflorescences increased or decreased. Therefore, inflorescence biomass and absolute nutrient accumulation should also be considered.

### 3.3. Transcriptomic Responses to KH_2_PO_4_ Treatment and Their Associations with Physiological Traits

At the molecular level, transcriptomic analysis identified 8142 differentially expressed genes (DEGs) between CN and 2KF, including 3386 up-regulated and 4756 down-regulated genes. In CN, which received no phosphorus or potassium fertilizer, the phosphate-starvation-associated transcription factors PHR2 and NRG2 and other phosphate-starvation-responsive genes exhibited relatively high expression, whereas potassium transport-related genes, including HAK and *kup*, showed reduced expression. In contrast, under 2KF, which received a more adequate supply of KH_2_PO_4_, the phosphate transporter genes PHT and PHO84 and the potassium transport-related gene *kup* showed up-regulation trends, accompanied by lower expression of phosphate-starvation-responsive genes (Figure 11 and Figure 14). The P–K interaction models proposed by [16] provide a potential framework for interpreting these expression patterns. Under phosphorus deficiency, insufficient intracellular inositol pyrophosphate InsP8 may limit the formation of SPX–PHR complexes, allowing PHR transcription factors to activate phosphate-starvation-responsive genes. This signaling response may also be associated with changes in potassium transport regulation. When phosphorus availability increases, InsP8-dependent SPX–PHR interactions may suppress phosphate-starvation signaling, potentially allowing the expression of nutrient transport-related genes to recover. Thus, the present transcriptomic results suggest that insufficient KH_2_PO_4_ supply may be associated with phosphate-starvation signaling and reduced expression of certain potassium transport-related genes, whereas more adequate KH_2_PO_4_ supply is associated with increased expression of genes involved in both phosphorus and potassium transport. These findings provide molecular-level insights into the differences in physiological characteristics and flowering performance between CN and 2KF.

It should be noted that KH_2_PO_4_ was the sole source of both phosphorus and potassium in this study. Therefore, changing the KH_2_PO_4_ application rate simultaneously altered the supply of both nutrients. The observed phenotypic, physiological, and transcriptomic differences should consequently be interpreted as the combined effects of KH_2_PO_4_ application rather than as the independent contributions of phosphorus and potassium. Because separate phosphorus and potassium treatments were not included, their individual regulatory effects cannot be quantitatively distinguished, nor can a causal synergistic or antagonistic relationship between P- and K-signaling pathways be established. Previous transcriptomic studies of *H. paniculata* have mainly focus on flowering-related hormonal regulation and aluminum-stress responses, with limited attention to nutrient interactions involving KH_2_PO_4_ [14,15]. By comparing CN and 2KF, the present study provides preliminary transcriptomic insights into this research area. However, as the transcriptomic analysis was limited to leaf samples collected at full bloom, the spatial and temporal specificity of the responses to KH_2_PO_4_ remains unclear and requires further investigation using multiple tissues and developmental stages.

## 4. Materials and Methods

### 4.1. Plants, Soil Preparation and Experiment Design

All experiments were conducted from April to September 2025 in a horticultural greenhouse at Sichuan Agricultural University, China. Greenhouse environmental conditions remained relatively stable throughout the experimental period. The average daytime temperature ranged from approximately 20 °C to 35 °C, and the relative humidity generally fluctuated between 50% and 85%. All treatments received the same routine cultivation management, including irrigation, weed control, and pest and disease management, to minimize differences in the growing conditions among plants. Based on the growth and developmental progression of *Hydrangea paniculata ‘Limelight’*, the experimental period was divided into five phenological stages: vegetative growth, flower bud differentiation, visible bud, full flowering, and late flowering. Measurements and sample collection were scheduled according to these developmental stages.

Uniformly grown one-year-old *Hydrangea paniculata ‘Limelight’* cuttings without any visible pest infestation or disease lesions were chosen as experimental materials, which could reduce the interference of individual plant differences on experimental outcomes.

The growing medium consisted of peat, perlite, and vermiculite at a volume ratio of 3:1:1. Each pot was filled with 2.0 kg of air-dried substrate. Several key physicochemical attributes were measured for the cultivation substrate. Before the experiment, the substrate had a pH of 7.41, an electrical conductivity (EC) of 654 μS cm^−1^, a bulk density of 0.765 g cm^−3^, and total porosity of 71.13%, alkali-hydrolyzable nitrogen of 337.5 mg kg^−1^, available phosphorus of 24.32 mg kg^−1^, available potassium 6.69 mg kg^−1^, and organic matter content of 411.41 g kg^−1^.

A two-factor experimental design was adopted. The first factor was the basal phosphorus supply gradient in the substrate, with KH_2_PO_4_ (Sinopharm Chemical Reagent Co., Ltd., Beijing, China) used as the combined phosphorus and potassium source. Four basal phosphorus levels were established: P1 (50 mg P_2_O_5_ kg^−1^ substrate); P2 (100 mg P_2_O_5_ kg^−1^ substrate); P3 (150 mg P_2_O_5_ kg^−1^ substrate); and P4 (200 mg P_2_O_5_ kg^−1^ substrate). Because KH_2_PO_4_ was used as the fertilizer source, potassium was supplied simultaneously according to the corresponding stoichiometric ratio. The second factor was whether foliar fertilization was applied at the bud stage, with two levels: no foliar spray (−KF) and 0.1% KH_2_PO_4_ foliar spray (+KF). The foliar treatment was applied once per week for two consecutive weeks. To distinguish the combined treatments, the abbreviations 1KF, 2KF, 3KF, and 4KF were used to represent P1 + KF, P2 + KF, P3 + KF, and P4 + KF, respectively, in all subsequent figures and tables.

In addition to the eight combined fertilization treatments generated by the two factors, two additional controls were included: a blank control (CK), without nitrogen, phosphorus, or potassium application, and a nitrogen-only control (CN), which received 100 mg N kg^−1^ substrate without P or K supply. All phosphorus–potassium fertilization treatments received a basal application of urea at 100 mg N kg^−1^ substrate to maintain a consistent nitrogen background and eliminate the confounding effect of nitrogen availability [12]. Each treatment consisted of 15 pots, giving a total of 150 pots. The eight factorial treatment combinations received the corresponding basal KH_2_PO_4_ application rates, with or without foliar KH_2_PO_4_ application at the visible bud stage. CK and CN were included as separate controls and were not part of the 4 × 2 factorial design. The experimental unit was an individual pot, and all pots were arranged randomly under the same cultivation conditions. The complete treatment design is shown in Table 8.

Basal fertilizer was applied once before transplanting. Fertilizers were dissolved in a specific volume of water and evenly incorporated into the growing medium, ensuring uniform distribution while avoiding leaching loss by controlling the water content to reach the substrate’s maximum water-holding capacity without drainage. Foliar fertilization at the bud stage was conducted in the early morning. Both the adaxial and abaxial leaf surfaces were sprayed evenly until fully wetted but not dripping. Throughout the experimental period, all treatments were maintained under identical irrigation, weeding, and pest control practices, and environmental conditions were kept as consistent as possible.

**Table 8:** Experimental treatment design.

Treatment	Base Fertilizer Phosphorus Level (mg P_2_O_5_/kg Matrix)	Base Fertilizer KH_2_PO_4_ Dosage (g/pot)	Bud Stage Topdressing	Urea Dosage (g/pot)
CK	0	0	None	0
CN	0	0		0.43
P1	50	0.19		
P2	100	0.38		
P3	150	0.57		
P4	200	0.76		
P1 + KF (1KF)	50	0.19	0.1% KH_2_PO_4_ foliar spray (once a week, for a total of two times)	
P2 + KF (2KF)	100	0.38		
P3 + KF (3KF)	150	0.57		
P4 + KF (4KF)	200	0.76		

Note: P1~P4 represent four gradient KH_2_PO_4_ base fertilizer treatments; 1KF, 2KF, 3KF, and 4KF correspond to P1 + KF, P2 + KF, P3 + KF, and P4 + KF compound topdressing treatments, respectively; CK is the no-fertilizer control, and CN is the nitrogen-only control; 0.1% KH_2_PO_4_ topdressing is sprayed once a week, with two consecutive sprays.

### 4.2. Determination of Vegetative Growth and Flowering Quality

Plant height, stem diameter, and other aboveground vegetative growth parameters were measured 42 days after basal fertilization.

At the late flowering stage, whole plants were harvested and separated into roots, stems, leaves, and flowers. Samples were first oven-treated at 105 °C for 30 min to inactivate enzymes and were then dried at 80 °C to constant weight. The dry mass of each organ was recorded, and total biomass and the root–shoot ratio were calculated [28].

A root scanning (CSX530, Graphtec Corporation, Yokohama, Japan) apparatus was utilized to quantify root morphological traits covering total root length, root surface area, root volume and mean root diameter [29,30]. Three independently potted plants were randomly selected from the 15 plants in each treatment, with each plant serving as one biological replicate (n = 3). The dates of visible bud emergence, first flowering, full flowering, and the end of flowering were recorded throughout the experimental period to determine the total flowering duration of each plant. Inflorescence diameter, individual floret diameter, and the number of inflorescences per plant were measured at full flowering [14]. Five plants were randomly selected from each treatment, and three inflorescences were measured per plant. The measurements were averaged for subsequent analysis.

### 4.3. Determination of Photosynthetic and Physiological Indicators

At the full flowering stage (10–15 August 2025), healthy, fully expanded, functional leaves from the middle canopy were collected, with three biological replicates per treatment. Samples were immediately frozen in liquid nitrogen and stored at −80 °C for subsequent physiological analyses.

Photosynthetic pigment contents were determined using the acetone–ethanol (acetone and ethanol, Sinopharm Chemical Reagent Co., Ltd., Beijing, China) extraction method [31]. Absorbance was measured with a UV–visible spectrophotometer (Ultrospec6300, GE Healthcare, Little Chalfont, UK), and chlorophyll and carotenoid contents were calculated accordingly. Gas exchange parameters were measured between 09:00 and 11:00 on sunny days using a LI-6400XT portable photosynthesis system (LI-COR Biosciences, Lincoln, NE, USA). The photosynthetic indexes determined in this experiment covered net photosynthetic rate (Pn), stomatal conductance (Gs), intercellular CO_2_ concentration (Ci), as well as transpiration rate (Tr). A portable fluorometer (MINI-PAM-II, Heinz Walz GmbH, Effeltrich, Germany) was employed to detect chlorophyll fluorescence indicators, which consisted of initial fluorescence (Fo), maximum fluorescence (Fm), maximum quantum yield of photosystem II (Fv/Fm) and potential photochemical activity (Fv/Fo) [32,33].

All physiological indicators were determined using healthy functional leaves collected from the same relative leaf position. Three biochemical indicators were detected through distinct colorimetric approaches: anthrone (Sinopharm Chemical Reagent Co., Ltd., Beijing, China) colorimetry for soluble sugar, Coomassie brilliant blue (Sinopharm Chemical Reagent Co., Ltd., Beijing, China) staining for soluble protein, and thiobarbituric acid (TBA) (Sinopharm Chemical Reagent Co., Ltd., Beijing, China) colorimetry for malondialdehyde (MDA). Peroxidase (POD) activity was determined using the guaiacol (Sinopharm Chemical Reagent Co., Ltd., Beijing, China) method, catalase (CAT) activity using the ultraviolet absorption method, and superoxide dismutase (SOD) activity using the nitro blue tetrazolium (NBT) (Sinopharm Chemical Reagent Co., Ltd., Beijing, China) method [34].

### 4.4. Determination of Nutrient Contents in Different Plant Organs

Root, stem, leaf, and flower samples were collected at the late flowering stage. Samples were first oven-dried at 105 °C for 30 min to inactivate enzymes and were then dried at 80 °C to a constant weight; the samples were ground and passed through a sieve for nutrient analysis. Root, stem, leaf, and flower samples (approximately 5 g of dry weight per organ per biological replicate) were collected at the late flowering stage. Plant materials were digested using the H_2_SO_4_–H_2_O_2_ (H_2_SO_4_ and H_2_O_2_, Sinopharm Chemical Reagent Co., Ltd., Beijing, China) digestion method to prepare analytical solutions. The Kjeldahl procedure was adopted to detect total nitrogen content [35]. As for total phosphorus, its concentration was assayed via the molybdenum–antimony colorimetry approach. An inductively coupled plasma optical emission spectrometer (ICP-OES, EXPEC 6500, Focused Photonics Inc., Hangzhou, China; serial number: 20242678S, Analysis and Testing Center of Sichuan Agricultural University) was utilized to quantify total potassium levels.

Based on organ dry mass and nutrient concentrations, N, P, and K accumulation in the whole plant and their distribution among roots, stems, leaves, and flowers were calculated [36,37].

### 4.5. Transcriptome Sequencing and qRT-PCR Validation

Based on the phenotypic and physiological results, the nitrogen-only control CN and the optimal fertilization treatment 2KF, corresponding to P2 + KF, were selected for transcriptome analysis. Functional leaves were collected at the full flowering stage, 63 days after basal fertilization [15]. Samples were immediately frozen in liquid nitrogen and sent to Shanghai Lingen Biotechnology Co., Ltd. (Shanghai, China) for total RNA extraction, library construction, and Illumina high-throughput sequencing.

Differentially expressed genes (DEGs) were identified using the thresholds |log_2_ fold change| ≥ 1 and adjusted *p* < 0.05. Genes related to phosphorus and potassium transport, phosphorus starvation response, carbon metabolism, photosynthesis, and stress signaling pathways were annotated. Gene Ontology (GO) and Kyoto Encyclopedia of Genes and Genomes (KEGG) enrichment analyses were then performed to characterize gene functions and regulatory pathways [38].

For qRT-PCR validation, we selected twelve key DEGs and adopted β-actin as the housekeeping reference gene. Primer Premier 5.0 (Premier Biosoft, San Francisco, CA, USA) was used to design gene-specific primers [15] and were manufactured by Tsingke Biotechnology Co., Ltd. (Beijing, China), with detailed sequences summarized in Table 9. Relative transcript abundance was quantified via the 2^−ΔΔCt^ approach. Every treatment contained three independent biological samples, each measured in three technical replicates. The consistency between qRT-PCR results and transcriptome data was used to evaluate the reliability of the sequencing results.

### 4.6. Statistical Analysis

All experimental data were organized and calculated using Microsoft Excel 2021 (Microsoft Corporation, Redmond, WA, USA), and statistical analyses were performed using SPSS 26.0 (IBM, Armonk, NY, USA). To avoid confounding statistical comparisons across different experimental groups, the data were analyzed according to the following procedures.

For the factorial treatments (P1–P4 × foliar spray): a two-way analysis of variance (ANOVA) was performed to evaluate the main effects of basal KH_2_PO_4_ application rate, foliar spray, and their interaction. When the interaction effect was significant, simple effects analysis was conducted.

For the control groups (CK and CN), an independent samples *t*-test was used to compare the two groups.

One-way ANOVA followed by Dunnett’s post hoc test was used to compare basal KH_2_PO_4_ application rates under non-foliar (P1–P4) and foliar application conditions (1KF–4KF), with each treatment compared against its respective control. In addition, two-way ANOVA was performed to evaluate the main effects of basal KH_2_PO_4_ application rate and foliar application, as well as their interaction effect, on the measured variables.

To compare treatments with and without foliar application at the same basal P level (e.g., P1 vs. 1KF), independent-samples *t*-tests were performed separately for each basal P level.

Pearson correlation analysis was used to assess relationships among indicators. Principal component analysis (PCA) was used for comprehensive evaluation of growth, physiological, flowering, and nutrient indicators to screen the optimal fertilization regime. Graphs were generated using Origin 2019 (OriginLab Corporation, Northampton, MA, USA). Data are presented as mean ± standard deviation (SD) [39]. Differences were considered statistically significant at *p* < 0.05.

## 5. Conclusions

Basal KH_2_PO_4_ application combined with foliar spraying at the budding stage differentially affected the physiological characteristics, nutrient accumulation and allocation, and flowering performance of *Hydrangea paniculata ‘Limelight’*. Among the treatments, 2KF (100 mg P_2_O_5_ kg^−1^ substrate as basal fertilizer plus a 0.1% KH_2_PO_4_ foliar spray) exhibited comparatively favorable photosynthetic performance, antioxidant status, nutrient allocation to inflorescences, and ornamental traits, including larger inflorescences and a longer orn·amental period. P1 and P4 showed imbalances among pigment accumulation, carbon assimilation, and photochemical efficiency, rather than uniformly lower performance, suggested that flowering performance depended not only on nutrient supply but also on nutrient allocation and physiological responses. Transcriptomic analysis identified 8142 differentially expressed genes between CN and 2KF, primarily associated with nutrient transport and homeostasis, carbon metabolism, and stress responses. The expression patterns of phosphate-starvation-responsive and P- and K-transport-related genes were consistent with the observed physiological and nutrient allocation differences, providing molecular-level insights into plant responses to KH_2_PO_4_ supply. The qRT-PCR results further supported the expression trends of selected candidate genes, although their specific functions and regulatory roles require further validation. Overall, under the conditions of this experiment, the 2KF treatment showed comparatively favorable performance, providing a reference for KH_2_PO_4_ fertilization management in *Hydrangea paniculata ‘Limelight’*; however, its optimal application rate and effectiveness under different cultivation conditions require further investigation.

## Figures and Tables

**Table 1:** Effects of different KH_2_PO_4_ concentrations on vegetative growth of *H. paniculata ‘Limelight’*.

Treatment	Stem Diameter (mm)	Leaf Length (mm)	Leaf Width (mm)	Crown Diameter (cm)	Plant Height (cm)	Number of Branches	Branch Length (cm)
Control groups							
CK	8.96 ± 1.14	81.97 ± 10.37	46.36 ± 4.59	49.66 ± 7.52	49.93 ± 5.35	8.67 ± 3.78	22.06 ± 3.93
CN	9.30 ± 0.69	72.71 ± 5.32	36.73 ± 2.34	54.16 ± 3.86	48.30 ± 5.99	5.33 ± 2.08	20.96 ± 2.78
Basal KH_2_PO_4_ gradients							
P1	8.81 ± 0.96 b	77.67 ± 3.06 a	39.60 ± 1.27 a	43.33 ± 1.62 a	44.53 ± 4.69 a	5.67 ± 3.05 a	25.40±2.91 a
P2	9.23 ± 0.47 b	81.97 ± 2.82 a	35.37 ± 2.75 a	43.76 ± 3.04 a	52.26 ± 5.50 a	4.67 ± 1.52 ab	26.46±4.88 a
P3	8.97 ± 0.12 b	81.74 ± 3.75 a	42.19 ± 3.42 a	41.13 ± 6.5 a	54.93 ± 5.31 a	1.33 ± 1.15 b	19.36±4.45 a
P4	11.15 ± 0.51 a	82.62 ± 3.48 a	40.60 ± 2.13 a	38.83 ± 4.34 a	44.53 ± 0.94 a	2.67 ± 1.15 ab	21.23±1.87 a

Note: Data were collected 42 days after basal fertilization, prior to bud-stage foliar KH_2_PO_4_ topdressing, which only reflects the independent effects of four basal KH_2_PO_4_ gradients on vegetative growth. CK = control without N, P, and K application; CN = nitrogen-only control; P1–P4 represent four different basal KH_2_PO_4_ concentrations. No significant differences were observed between CK and CN (*p* > 0.05). For P1–P4, different lowercase letters within the same column indicate significant differences among treatments (*p* < 0.05), while no letters indicate no significant differences.

**Table 2:** Effect of Different KH_2_PO_4_ Concentrations and Fertilization Methods on Biomass.

Treatment	Root Biomass (g)	Stem Biomass (g)	Leaf Biomass (g)	Total Biomass (g)	Ratio
Control groups					
CK	25.07 ± 6.80	17.42 ± 5.90	4.86 ± 2.14	48.57 ± 9.44	1.13 ± 0.27
CN	18.09 ± 4.07	11.97 ± 1.76	7.04 ± 2.97	37.37 ± 3.78	0.98 ± 0.38
Basal					
P1	17.21 ± 5.12 a	11.58 ± 4.68 a	4.30 ± 0.94 a	34.24 ± 7.82 a	1.11 ± 0.32 a
P2	13.19 ± 4.81 a	8.31 ± 1.60 a	2.29 ± 1.98 a	25.05 ± 8.41 a	1.22 ± 0.16 a
P3	13.40 ± 3.30 a	7.96 ± 2.39 a	2.28 ± 1.71 a	25.99 ± 6.22 a	1.37 ± 0.42 a
P4	16.65 ± 2.14 a	9.37 ± 0.08 a	3.65 ± 2.41 a	31.29 ± 8.21 a	1.33 ± 0.26 a
Fertilization during the bud					
1KF	15.84 ± 2.57 B	7.49 ± 0.99 B	2.95 ± 2.86 B	26.99 ± 5.25 B	1.52 ± 0.14 A
2KF	15.44 ± 2.52 B	7.99 ± 2.55 B	2.68 ± 2.61 B	27.36 ± 6.71 B	1.56 ± 0.48 A
3KF	15.16 ± 2.48 B	8.14 ± 2.28 B	3.71 ± 2.46 B	27.70 ± 6.86 B	1.39 ± 0.47 A
4KF	32.29 ± 2.36 A	21.02 ± 4.97 A	9.47 ± 5.33 A	63.34 ± 10.05 A	1.13 ± 0.39 A
Variation source					
Base fertilizer concentration	***	***	*	***	ns (0.802)
Fert	***	ns (0.104)	ns (0.127)	*	ns (0.272)
Base fertilizer concentration × Fert	***	***	ns (0.096)	***	ns (0.300)

Note: Data in the table are presented as mean ± standard error. Different lowercase letters within the same column indicate significant differences among basal fertilizer phosphorus-gradient treatments at the *p* < 0.05 level, whereas different uppercase letters indicate significant differences between bud-emergence-stage topdressing treatments at the *p* < 0.05 level. *** indicates *p* < 0.001, ** indicates *p* < 0.01, * indicates *p* < 0.05, and ns indicates no significant difference (*p* > 0.05).

**Table 3:** Two factor ANOVA of the Effects of Different KH_2_PO_4_ Concentrations and Fertilization Methods on Root Morphology.

Treatment	Root Length (cm)	Root Surface Area (cm^2^)	Root Volume (cm^3^)	Root Diameter (mm)
Control groups				
CK	24.17 ± 0.61	536.45 ± 18.73	5482.24 ± 660.95	6.12 ± 1.54
CN	30.93 ± 5.69	540.54 ± 84.31	5295.32 ± 1507.52	5.27 ± 1.20
Base fertilizer concentration				
P1	26.30 ± 8.63 b	419.87 ± 31.01 a	1457.16 ± 1030.37 a	6.70 ± 1.22 a
P2	51.35 ± 5.87 a	279.35 ± 36.90 b	120.86 ± 85.46 a	4.33 ± 0.17 a
P3	43.35 ± 13.08 ab	415.47 ± 13.53 a	498.52 ± 1149.59 a	5.53 ± 1.26 a
P4	28.95 ± 0.21 ab	295.12 ± 79.79 ab	941.68 ± 332.93 a	6.29 ± 0.04 a
Fertilization during the bud stage				
1KF	32.40 ± 4.95 AB	351.15 ± 0.85 A	2756.38 ± 117.49 AB	5.59 ± 0.01 C
2KF	26.40 ± 2.55 B	323.99 ± 63.29 A	3362.16 ± 94.93 A	6.40 ± 0.16 BC
3KF	34.90 ± 1.98 B	282.62 ± 49.41 A	2068.17 ± 578.01 B	6.47 ± 0.59 B
4KF	25.10 ± 0.28 B	264.02 ± 9.22 A	2826.81 ± 554.29 AB	8.07 ± 0.12 A
Variation source				
Base fertilizer concentration	*	*	ns (0.678)	*
Fert	*	ns (0.065)	ns (0.156)	*
Base fertilizer concentration × Fert	*	ns (0.105)	ns (0.188)	*

Note: Data in the table are presented as mean ± standard error. Different lowercase letters within the same column indicate significant differences among basal fertilizer phosphorus-gradient treatments at the *p* < 0.05 level, whereas different uppercase letters indicate significant differences between bud-emergence-stage topdressing treatments at the *p* < 0.05 level. * indicates *p* < 0.05, and ns indicates no significant difference (*p* > 0.05).

**Table 4:** Effects of Different KH_2_PO_4_ Concentrations and Fertilization Methods on the Flowering Period of *Hydrangea paniculata ‘LimeLight’*.

Group	Initial Flowering Stage	Full Bloom	Late Blooming Period	Flowering Period	Blooming Period Extension
CK	29 June 2025	10 July 2025	10 September 2025	73	−4
CN	11 July 2025	3 August 2025	26 September 2025	77	0
P1	1 July 2025	14 July 2025	23 September 2025	81	4
P2	29 June 2025	9 July 2025	22 September 2025	85	8
P3	2 July 2025	15 July 2025	26 September 2025	86	9
P4	6 July 2025	14 July 2025	25 September 2025	81	4
1KF	6 July 2025	18 July 2025	29 September 2025	85	8
2KF	2 July 2025	11 July 2025	30 September 2025	90	13
3KF	22 June 2025	12 July 2025	21 September 2025	91	14
4KF	19 July 2025	28 July 2025	16 October 2025	89	12

Note: The flowering period refers to the total number of days for a single group; flowering period extension refers to the increase in the number of blooming days compared to the control group (CN).

**Table 5:** Two-factor ANOVA of the Effects of Different KH_2_PO_4_ Concentrations and Fertilization Methods on Photosynthetic Pigments in Leaves.

Treatment	Chlorophyll a (mg/g)	Chlorophyll b (mg/g)	Total Chlorophyll (mg/g)	Carotenoid Content (mg/g)
Control groups				
CK	0.53 ± 0.16	0.24 ± 0.06	0.76 ± 0.22	1.79 ± 0.55
CN	0.53 ± 0.04	0.23 ± 0.01	0.76 ± 0.05	1.85 ± 0.17
Base fertilizer concentration				
P1	0.77 ± 0.15 a	0.34 ± 0.10 a	1.11 ± 0.24 a	2.48 ± 0.48 a
P2	0.39 ± 0.08 b	0.21 ± 0.04 b	0.60 ± 0.11 b	1.48 ± 0.23 b
P3	0.44 ± 0.04 b	0.21 ± 0.02 b	0.65 ± 0.05 b	1.54 ± 0.06 b
P4	0.35 ± 0.05 b	0.19 ± 0.03 b	0.54 ± 0.08 b	1.30 ± 0.23 b
Fertilization during the bud stage				
1KF	0.41 ± 0.10 AB	0.23 ± 0.06 AB	0.64 ± 0.13 AB	1.43 ± 0.29 AB
2KF	0.35 ± 0.11 B	0.18 ± 0.03 B	0.53 ± 0.14 B	1.19 ± 0.36 B
3KF	0.34 ± 0.07 B	0.18 ± 0.02 B	0.51 ± 0.09 B	1.20 ± 0.19 B
4KF	0.58 ± 0.08 A	0.28 ± 0.05 A	0.86 ± 0.13 A	1.94 ± 0.24 A
Variation source				
Base fertilizer concentration	***	***	***	***
Fert	*	ns (0.155)	*	**
Base fertilizer concentration × Fert	***	***	***	***

Note: Data in the table are presented as mean ± standard error. Different lowercase letters within the same column indicate significant differences among basal fertilizer phosphorus-gradient treatments at the *p* < 0.05 level, whereas different uppercase letters indicate significant differences between bud-emergence-stage topdressing treatments at the *p* < 0.05 level. *** indicates *p* < 0.001, ** indicates *p* < 0.01, * indicates *p* < 0.05, and ns indicates no significant difference (*p* > 0.05).

**Table 6:** Two-way ANOVA of the Effects of Different KH_2_PO_4_ Concentrations and Fertilization Methods on Leaf Photosynthetic Parameters.

Treatment	Pn (mmol m^−2^ s^−1^)	Tr (µmol m^−2^ s^−1^)	Ci (µmol mol^−1^)	Gs (mol m^−2^ s^−1^)
Control groups				
CK	1.74 ± 0.84	2.28 ± 0.16	407.09 ± 19.61	0.13 ± 0.07
CN	0.81 ± 0.24	1.98 ± 0.53	378.29 ± 24.59	0.05 ± 0.02
Base fertilizer concentration				
P1	0.37 ± 0.07 b	1.09 ± 0.45 b	408.61 ± 51.24	0.02 ± 0.01 b
P2	0.28 ± 0.14 b	1.40 ± 1.10 b	350.96 ± 60.75	0.02 ± 0.01 b
P3	1.27 ± 0.65 a	2.84 ± 0.16 a	383.93 ± 57.31	0.10 ± 0.05 a
P4	1.10 ± 0.52 ab	2.01 ± 0.39 ab	375.56 ± 62.68	0.08 ± 0.04 ab
Fertilization during the bud stage				
1KF	1.13 ± 0.59	2.27 ± 0.11	379.27 ± 34.63	0.08 ± 0.05
2KF	0.90 ± 0.17	2.53 ± 0.43	368.51 ± 2.17	0.06 ± 0.01
3KF	0.86 ± 0.17	2.85 ± 0.48	356.19 ± 11.14	0.06 ± 0.01
4KF	0.69 ± 0.54	2.18 ± 0.61	301.91 ± 130.11	0.04 ± 0.04
Variation source				
Base fertilizer concentration	ns (0.267)	*	ns (0.456)	ns (0.269)
Fert	ns (0.421)	*	ns (0.247)	ns (0.547)
Base fertilizer concentration × Fert	*	ns (0.175)	ns (0.625)	ns (0.053)

Note: Data in the table are presented as mean ± standard error. Different lowercase letters within the same column indicate significant differences among basal fertilizer phosphorus-gradient treatments at the *p* < 0.05 level, whereas different uppercase letters indicate significant differences between bud-emergence-stage topdressing treatments at the *p* < 0.05 level. *** indicates *p* < 0.001, ** indicates *p* < 0.01, * indicates *p* < 0.05, and ns indicates no significant difference (*p* > 0.05).

**Table 7:** Two-way ANOVA of the Effects of Different KH_2_PO_4_ Concentrations and Fertilization Methods on Chlorophyll Fluorescence Content.

Treatment	Fo	Fm	Fv/Fo	Fv/Fm
Control groups				
CK	165.22 ± 55.87	653.33 ± 121.89	3.10 ± 0.58	0.75 ± 0.05
CN	154.11 ± 64.49	551.67 ± 248.66	2.62 ± 0.62	0.72 ± 0.06
Base fertilizer concentration				
P1	176.56 ± 123.18	593.11 ± 423.51	2.37 ± 0.92	0.68 ± 0.10
P2	182.89 ± 69.31	662.33 ± 340.78	2.63 ± 0.94	0.70 ± 0.09
P3	141.22 ± 35.73	545.67 ± 138.89	2.88 ± 0.47	0.74 ± 0.03
P4	150.44 ± 35.64	513.11 ± 159.92	2.38 ± 0.52	0.70 ± 0.04
Fertilization during the bud stage				
1KF	133.89 ± 26.07 B	426.22 ± 105.26 B	2.20 ± 0.56 BC	0.68 ± 0.06 B
2KF	118.89 ± 32.41 B	508.67 ± 179.19 AB	3.29 ± 1.09 A	0.76 ± 0.05 A
3KF	170.44 ± 50.84 A	642.56 ± 215.2 A	2.80 ± 0.68 AB	0.73 ± 0.06 AB
4KF	135.11 ± 30.37 B	360.33 ± 83.29 B	1.71 ± 0.51 C	0.62 ± 0.06 C
Variation source				
Base fertilizer concentration	ns (0.904)	ns (0.158)	***	**
Fert	ns (0.100)	ns (0.092)	ns (0.719)	ns (0.525)
Base fertilizer concentration × Fert	ns (0.109)	ns (0.269)	ns (0.074)	*

Note: Data in the table are presented as mean ± standard error. Different lowercase letters within the same column indicate significant differences among basal fertilizer phosphorus-gradient treatments at the *p* < 0.05 level, whereas different uppercase letters indicate significant differences between bud-emergence-stage topdressing treatments at the *p* < 0.05 level. *** indicates *p* < 0.001, ** indicates *p* < 0.01, * indicates *p* < 0.05, and ns indicates no significant difference (*p* > 0.05).

**Table 9:** Information on RT-qPCR primer sequences.

Gene ID	Forward Primer (5′–3′)	Reverse Primer (5′–3′)
β-actin (housekeeping gene)	GCCTGCCATGTATGTTGCCATC	CGGAATCCAGCACAATACCAGTTG
TRINITY_DN45181_c0_g1	TGCCGAGGTACGACAACT	TTCTTCACAAACGCCACA
TRINITY_DN48444_c2_g1	CCTCAACCGCCTACCCTT	CAGTCTCCTGTTCAGCACC
TRINITY_DN50466_c1_g1	AGCAGGCATCAAGTTTGT	TTGCTCGCTGACTTCACT
TRINITY_DN37755_c0_g3	ACGGTGGTCAACAGAGCG	AAGAAAGCAACGGGAGGC
TRINITY_DN42240_c0_g1	GGAATTATTGGACCTGACC	GGCTGGGATTGAAGAGTG
TRINITY_DN35931_c0_g1	GGGAACAAATCCACAGAC	CCATACTCGCAAAGCAGA
TRINITY_DN51823_c2_g1	CACCACAGCAATCCCAACG	AAGAACCGGGTCCGCAAA
TRINITY_DN35421_c0_g1	TCCAAGGTCCAGAAACAT	CAGTCTAGTACGGCGATAA
TRINITY_DN34930_c0_g1	TCAGGGAAGAGCGTTATA	AGAGTGAGAAGACCGAGAC
TRINITY_DN46143_c0_g1	AAATCAAGCCATCCTGTC	CGTTTGTAGCGTGAGAAC
TRINITY_DN38919_c0_g1	CAACTGGGTTCGTAATCTG	GTGCGTCTCGTCTTCTTT
TRINITY_DN47954_c0_g1	GAACCCAAAGGGAAACCA	GGCCTCGGATGAACCAAC

## Data Availability

The data presented in this study are available on request from the corresponding author.
